# Selective Thrombin Modulation by AYA1809002: Stability and On-Demand Reversal of an Exosite I-Targeting Aptamer

**DOI:** 10.3390/ph19091431

**Published:** 2026-09-10

**Authors:** Mohamad Ammar Ayass, Natalya Griko, Victor Pashkov, Jin Zhang, Ghulam Abbas, Tutku Okyay, Kevin Zhu, Lina Abi-Mosleh

**Affiliations:** Ayass Bioscience LLC, 8501 Wade Blvd, Bldg 9, Frisco, TX 75034, USA

**Keywords:** aptamer, anticoagulation, thrombin, Exosite I, reverse-complement oligonucleotide

## Abstract

**Background**: Thrombin is a central mediator of coagulation and platelet activation and a key anticoagulant target. Direct thrombin inhibitors broadly suppress thrombin proteolytic activity, potentially disrupting procoagulant and anticoagulant signaling. Exosite-targeting aptamers may enable selective modulation and programmable reversal. **Methods:** We characterize AYA1809002, a 40-nt DNA aptamer targeting thrombin Exosite I. **Results:** AYA1809002 bound thrombin with nanomolar affinity and inhibited fibrin formation with an IC_50_ of 25.7 nM and K_*i*_ of 10.6 nM. Reduced γ-thrombin binding supported an Exosite I-dependent mechanism. AYA1809002 preserved thrombin activity toward small peptide substrates and thrombomodulin-dependent protein C activation. It showed no measurable activity against tested coagulation, anticoagulant, fibrinolytic, and control enzymes and inhibited thrombin-mediated platelet activation while preserving responses to non-thrombin agonists. AYA1809002 showed greater functional stability than thrombin-binding aptamer NU172 following serum exposure and remained active under thermal, pH, oxidative, and photostability stress. Anticoagulant activity was rapidly and sequence-specifically reversed by a reverse-complement oligonucleotide, while lipid conjugation preserved function. AYA1809002 also retained high-affinity binding to rat thrombin and anticoagulant activity in rat plasma. **Conclusions:** These findings identify AYA1809002 as a selective, stable, reversible Exosite I-targeting thrombin aptamer that suppresses key procoagulant functions while preserving thrombomodulin-dependent protein C activation.

## 1. Introduction

Thromboembolic disorders are a major cause of morbidity and mortality worldwide and contribute to conditions such as venous thromboembolism, stroke, myocardial infarction, and thrombotic complications associated with cardiovascular interventions. Thrombosis has been estimated to contribute to approximately one in four deaths globally, highlighting the critical importance of effective anticoagulant therapy [1]. Anticoagulants are widely used for prevention and treatment of thrombotic disease and for procedural anticoagulation. However, current therapies remain constrained by a narrow therapeutic window in which effective thrombosis prevention is intrinsically associated with an increased risk of bleeding [2,3,4]. Major bleeding events remain a frequent complication of anticoagulant therapy and may require treatment interruption, hospitalization, or emergency reversal [3,4,5]. Consequently, there is substantial interest in anticoagulant strategies that provide effective thrombin suppression while allowing rapid and controllable reversal of activity. This need is particularly acute in high-risk settings such as cardiac surgery, acute coronary syndrome management, and venous thromboembolism treatment, where the consequences of both inadequate anticoagulation and uncontrolled bleeding are severe.

Thrombin is a central protease within the coagulation cascade and functions both as the terminal enzyme responsible for fibrin formation and as a regulator of coagulation amplification. In addition to converting fibrinogen to fibrin, thrombin activates upstream cofactors including factors V, VIII, and XI, thereby reinforcing thrombin generation through positive feedback mechanisms [6]. Thrombin also links coagulation to platelet activation through cleavage of protease-activated receptors (PAR-1 and PAR-4), promoting platelet aggregation and thrombus stabilization [7]. Because of this central regulatory role, thrombin has long been recognized as a key therapeutic target for anticoagulation.

Direct thrombin inhibitors such as dabigatran exert their anticoagulant effects by binding the catalytic active site of thrombin and blocking substrate cleavage [8,9]. Although highly effective, catalytic-site inhibition suppresses essentially all thrombin proteolytic activity and can interfere with physiological thrombin functions beyond fibrin formation. This broad inhibition may disrupt the balance between procoagulant and anticoagulant signaling pathways and contribute to bleeding complications in clinical use [6,10]. These limitations have stimulated interest in alternative strategies capable of selectively modulating thrombin activity rather than completely abolishing it.

Thrombin contains a catalytic active site and two positively charged regulatory domains known as Exosite I and Exosite II. Exosite I, often referred to as the fibrinogen-recognition site, mediates binding of fibrinogen, thrombomodulin, and protease-activated receptors, whereas Exosite II primarily functions as a heparin-binding domain interacting with glycosaminoglycans and other regulatory molecules [11,12,13]. These Exosites enable thrombin to recognize macromolecular substrates and regulatory cofactors and therefore represent attractive targets for selective modulation of thrombin function. Agents targeting Exosite I can interfere with fibrinogen recognition while preserving catalytic competence toward small substrates, enabling substrate-selective inhibition of thrombin activity. Experimental validation of Exosite I engagement is commonly performed using γ-thrombin, a proteolytically modified form of thrombin that retains catalytic activity but lacks an intact Exosite I surface.

Nucleic acid aptamers have emerged as a promising class of therapeutic molecules capable of targeting extended protein surfaces such as thrombin Exosites with high affinity and specificity. Aptamers are short single-stranded oligonucleotides that fold into defined three-dimensional structures and bind proteins through shape complementarity and electrostatic interactions [14,15,16]. A distinctive advantage of aptamer-based therapeutics is the ability to rapidly neutralize their activity using reverse-complement oligonucleotide that disrupt aptamer structure through hybridization, enabling rapid and sequence-specific reversal of pharmacological activity [15,17]. Such programmable reversibility is particularly attractive in anticoagulation, where rapid restoration of hemostasis may be required during bleeding events or surgical procedures. Several thrombin-binding aptamers have been described, including the clinically evaluated aptamer NU172, which targets thrombin Exosite I and has been investigated as a controllable anticoagulant in clinical studies [17,18,19]. However, broader clinical translation of thrombin aptamers has been limited by susceptibility to nuclease degradation, reduced stability in biological fluids, and incomplete mechanistic characterization of their interactions with thrombin and the coagulation cascade [15,20]. These challenges highlight the need for next-generation thrombin aptamers that combine high thrombin selectivity with improved biological stability and controllable pharmacology.

Recently, we reported the discovery of a series of high-affinity thrombin-binding DNA aptamers, including AYA1809002, identified through iterative selection and optimization for thrombin recognition [21]. In that study, AYA1809002 demonstrated nanomolar binding affinity to thrombin and produced dose-dependent anticoagulant activity in plasma and whole-blood assays. Importantly, its anticoagulant effects could be neutralized using reverse-complement (RC) oligonucleotides, demonstrating the feasibility of programmable anticoagulant control.

Building on these findings, the present study provides a mechanistic and functional characterization of AYA1809002. Using competitive binding studies, γ-thrombin binding analysis, enzymatic assays, and plasma coagulation testing, we demonstrate that AYA1809002 binds thrombin through an Exosite I-dependent mechanism while preserving catalytic activity toward small peptide substrates and selectively inhibiting fibrinogen cleavage. Direct biochemical profiling against a panel of coagulation, anticoagulant, fibrinolytic, and control enzymes demonstrates a high degree of thrombin selectivity. Importantly, AYA1809002 preserves thrombomodulin-dependent protein C activation, indicating that inhibition of thrombin-mediated fibrin formation does not abolish this endogenous anticoagulant pathway.

Functional studies further show inhibition of thrombin-mediated platelet activation while preserving responses to non-thrombin agonists. We additionally demonstrate rapid antidote-mediated reversal, improved stability relative to NU172, preservation of activity following lipid conjugation, and retention of binding to rat thrombin and anticoagulant activity in rat plasma. Collectively, these findings establish AYA1809002 as a highly selective, reversible Exosite I-targeting thrombin aptamer that suppresses key procoagulant functions while preserving thrombomodulin-dependent protein C activation.

## 2. Results

### 2.1. AYA1809002 Binds Human α-Thrombin with High Affinity

**Figure 1:**
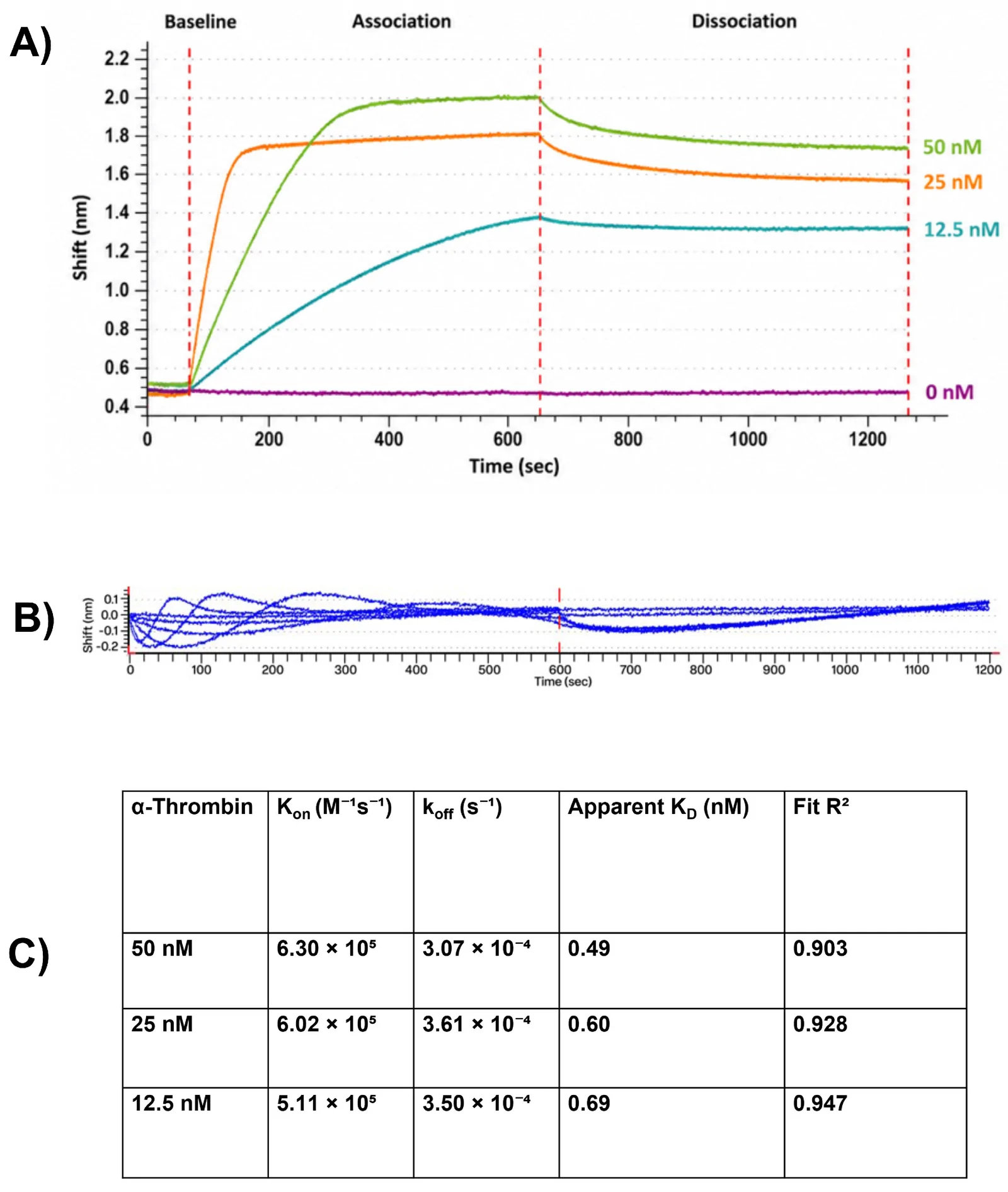
Biolayer interferometric analysis of AYA1809002 binding to human α-thrombin. (**A**) Representative BLI sensorgrams and fitted curves for human α-thrombin at 12.5, 25, and 50 nM, with a 0 nM reference control. Biotinylated AYA1809002 was immobilized on streptavidin biosensors, and association and dissociation were monitored for 600 s each. Sensorgrams were reference-subtracted and fitted using a 1:1 Langmuir interaction model in GatorOne software. (**B**) Residual plots corresponding to the fitted sensorgrams. (**C**) Apparent kinetic parameters and full-fit R^2^ values obtained from the kinetic analysis.

To quantitatively characterize the interaction between AYA1809002 and human α-thrombin, binding kinetics were determined by biolayer interferometry (BLI). Representative sensorgrams demonstrated concentration-dependent binding of AYA1809002 to human α-thrombin, with rapid association followed by slow dissociation (Figure 1). The 12.5–50 nM sensorgrams were fitted using a 1:1 Langmuir interaction model (Figure 1A), with full-fit R^2^ values of 0.903–0.947. Residual analysis is shown in Figure 1B. Kinetic analysis yielded apparent k_*on*_ values of 5.11–6.30 × 10^5^ M^−1^ s^−1^ and k_*off*_ values of 3.07–3.61 × 10^−4^ s^−1^, corresponding to apparent K_*D*_ values of 0.49–0.69 nM (Figure 1C). These findings demonstrate high-affinity binding of AYA1809002 to human α-thrombin and provide quantitative kinetic support for the subsequent mechanistic and functional studies.

### 2.2. AYA1809002 Binds Thrombin Through Exosite I and Selectively Inhibits Fibrinogen Recognition

**Figure 2:**
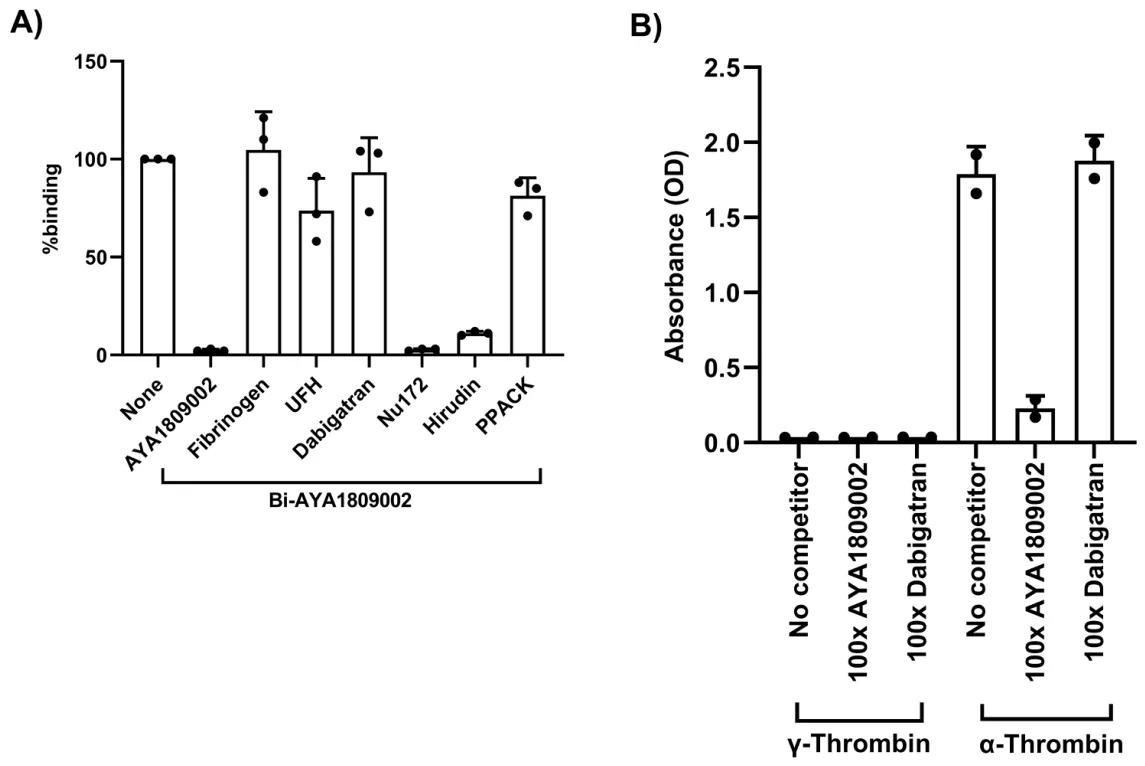
Competitive binding analysis identifies Exosite I as the primary AYA1809002 interaction site on thrombin. (**A**) Biotinylated AYA1809002 (Bi-AYA1809002) was incubated with immobilized human α-thrombin in the absence or presence of the indicated unlabeled competitors at 100-fold molar excess. (**B**) Binding of Bi-AYA1809002 to human α-thrombin and γ-thrombin was evaluated in the absence or presence of excess unlabeled AYA1809002 or dabigatran. Results are expressed relative to Bi-AYA1809002 alone (100%). All data were represented of at least two independent experiments. Error bars represented mean ± SD of independent experiment performed in triplicate.

To define the thrombin-binding mechanism of AYA1809002, competitive binding and functional assays were performed using ligands with well-characterized thrombin interaction sites (Figure 2). Biotinylated AYA1809002 bound strongly to immobilized human thrombin and was almost completely displaced by excess unlabeled aptamer, confirming binding specificity. The Exosite I ligands hirudin and NU172 strongly inhibited binding [15,22], whereas the catalytic-site inhibitors dabigatran and PPACK and the Exosite II ligand unfractionated heparin (UFH) produced little or no competition [8,11,13]. Notably, fibrinogen did not measurably displace Bi-AYA1809002 under the conditions of the competition assay, with aptamer binding remaining close to that observed in the absence of competitor. This finding indicates that fibrinogen does not efficiently displace thrombin-bound AYA1809002 in this surface-based assay but does not establish whether the two ligands occupy identical or fully overlapping contact sites within Exosite I [23]. In contrast, the strong displacement observed with the established Exosite I ligands NU172 and hirudin, together with the markedly reduced binding of AYA1809002 to γ-thrombin, provides complementary evidence that AYA1809002 recognition is centered on the Exosite I region. The functional consequence of this interaction was evaluated independently in fibrinogen-cleavage assays described below. Collectively, the competition profile supports a high-affinity thrombin interaction centered on Exosite I, with little evidence for direct engagement of the catalytic site or Exosite II (Figure 2A).

The requirement for an intact Exosite I surface was further evaluated using γ-thrombin, a proteolytically modified form of thrombin that retains catalytic activity but lacks a functional Exosite I binding surface. γ-Thrombin readily cleaved a fluorogenic substrate, confirming preservation of active-site function. As expected, dabigatran strongly inhibited γ-thrombin activity, whereas AYA1809002 had no measurable effect. In ELISA binding studies, AYA1809002 bound efficiently to α-thrombin but exhibited only background-level binding to γ-thrombin, providing independent evidence that aptamer recognition depends on an intact Exosite I surface [13,24,25,26] (Figure 2B).

**Figure 3:**
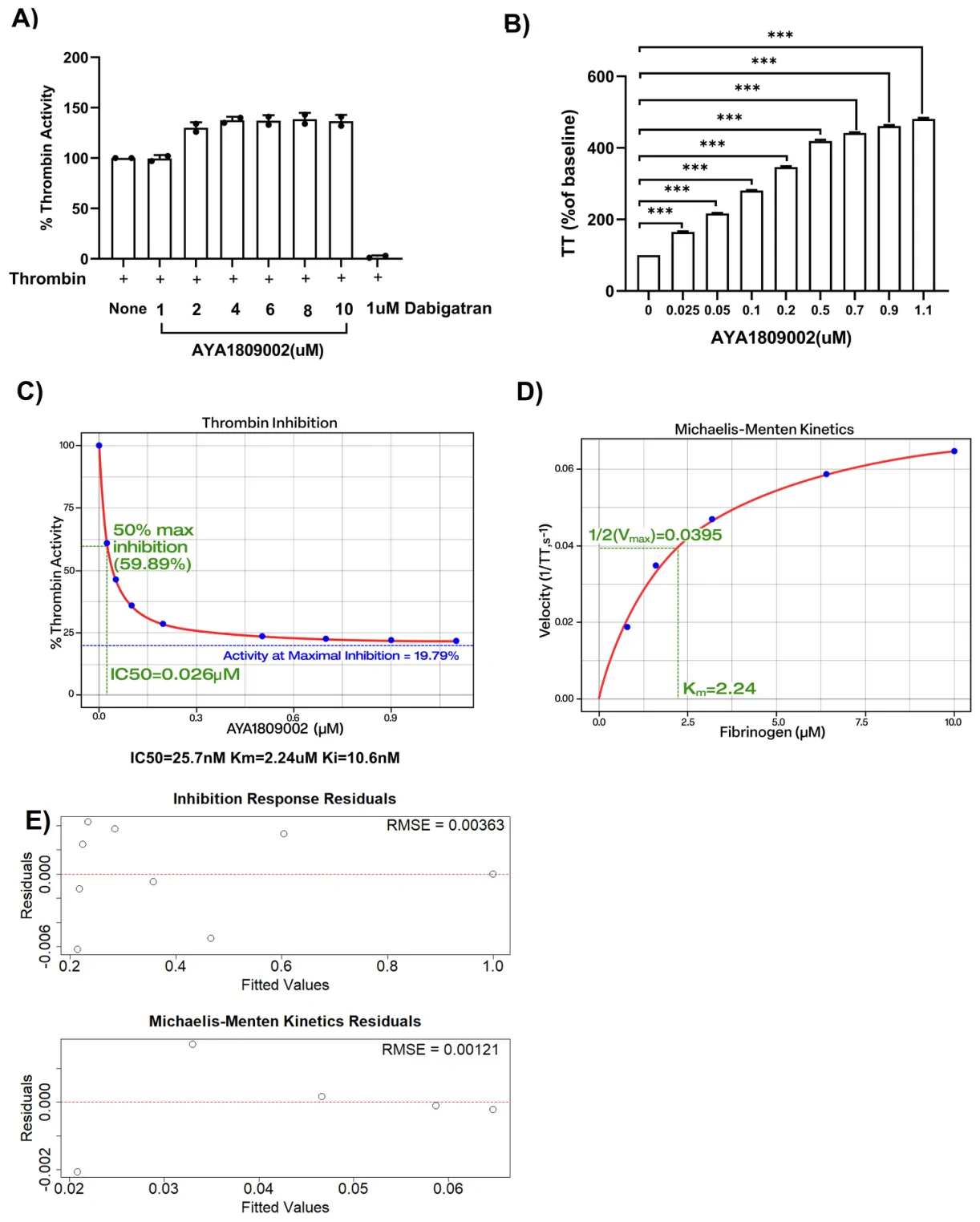
AYA1809002 selectively inhibits thrombin-mediated fibrin formation without blocking catalytic activity. (**A**) Thrombin activity toward a synthetic fluorogenic peptide substrate in the presence of AYA1809002 or dabigatran. All data were represented of two independent experiments. Error bars represented mean ± SD of independent experiment in triplicate. (**B**) Concentration-dependent inhibition of thrombin-mediated fibrin formation by AYA1809002 (IC_50_ = 25.7 nM). All data were represented of five independent experiments. Error bars represented mean ± SD of independent experiment in triplicate. Comparisons vs. 0-concentration was analyzed using repeat measurement test with Holm-adjusted Paired *t*-test (Supplementary Table S2). *** indicated *p* < 0.001 (**C**,**D**) Michaelis-Menten analysis of fibrinogen-dependent thrombin activity yielded an apparent Km of 2.24 µM and a calculated K_*i*_ of 10.6 nM. (**E**) Residual plots for the inhibition-response and Michaelis-Menten fits, with corresponding root mean square error (RMSE) values of 0.00363 and 0.00121, respectively.

To distinguish between catalytic-site inhibition and substrate-selective modulation, thrombin activity was evaluated using both a fluorogenic peptide substrate assay and a fibrinogen cleavage assay. AYA1809002 did not inhibit cleavage of a small fluorogenic substrate at concentrations up to 10 µM, whereas dabigatran potently suppressed substrate turnover [27] (Figure 3A). In contrast, AYA1809002 produced concentration-dependent inhibition of fibrin formation in a purified thrombin–fibrinogen system, with a functional IC_50_ of 25.7 nM (Figure 3B,C). The response increased progressively with increasing concentration, from 22.28 ± 0.15 at the zero-concentration control to 107.08 ± 0.11 at a concentration of 1.1 µM, corresponding to an approximately 4.8-fold increase. All tested concentrations (0.025–1.1 µM) produced significantly higher responses than the zero-concentration control after adjustment for multiple comparisons (Holm-adjusted *p* < 0.001 for all comparisons; Figure 3B; Supplementary Table S2). Analysis of thrombin time measurements obtained across increasing fibrinogen concentrations yielded an apparent fibrinogen Km of 2.24 µM and an estimated K_*i*_ of approximately 10.6 nM (Figure 3D). Residual plots for the inhibition-response and Michaelis–Menten fits are shown in Figure 3E, with corresponding root mean square error (RMSE) values of 0.00363 and 0.00121, respectively.

Together, these findings demonstrate that AYA1809002 binds thrombin through an Exosite I-dependent mechanism while preserving catalytic-site activity, resulting in potent and selective inhibition of fibrinogen-dependent thrombin function.

### 2.3. AYA1809002 Demonstrates a Thrombin-Centered Anticoagulant Phenotype with High Target Selectivity

**Figure 4:**
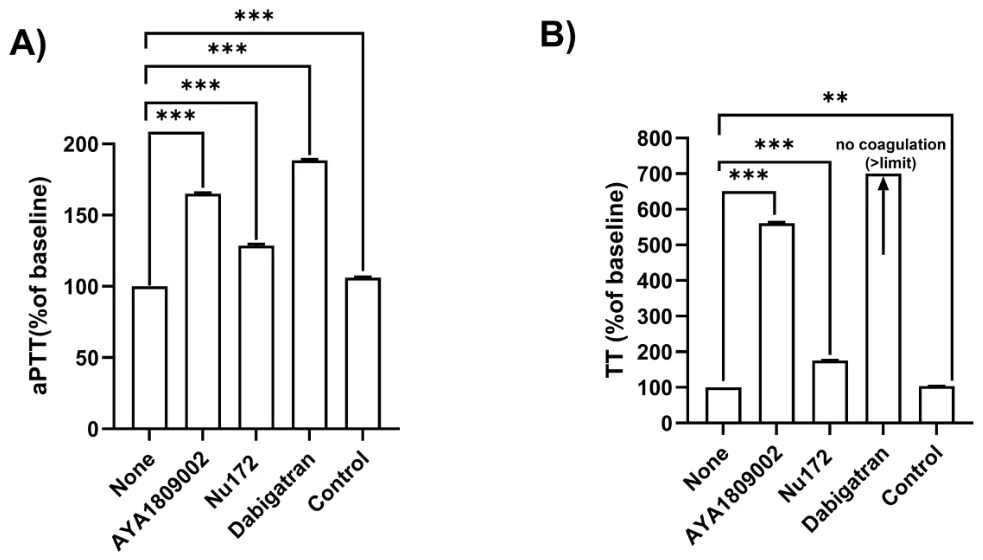
Plasma clotting assays demonstrate a thrombin-centered anticoagulant phenotype of AYA1809002. (**A**) Activated partial thromboplastin time (aPTT) and (**B**) thrombin time (TT) in citrated human plasma following treatment with AYA1809002, NU172, dabigatran, or non-binding control aptamer (1 µM). Results are expressed relative to untreated plasma (100%). Data represent mean ± SD from five independent experiments, each performed in triplicate. Overall differences were assessed using the Friedman test, followed by paired comparisons with the untreated control using two-sided paired *t*-tests with Holm adjustment *** indicated *p* < 0.0001; ** indicated *p* < 0.001 versus the corresponding control.

The anticoagulant profile of AYA1809002 was evaluated using plasma coagulation assays and biochemical selectivity studies. In citrated human plasma, AYA1809002 (1 µM) produced marked prolongation of thrombin time (TT) while exerting only moderate effects on activated partial thromboplastin time (aPTT) and minimal effects on prothrombin time (PT) (Figure 4). Compared with the clinically evaluated thrombin aptamer NU172, AYA1809002 produced substantially greater TT prolongation, whereas dabigatran completely suppressed clot formation under the same assay conditions. These findings indicate that AYA1809002 produces a thrombin-centered anticoagulant phenotype consistent with selective inhibition of thrombin-mediated fibrin formation.

To evaluate target specificity, AYA1809002 was tested against a panel of coagulation, anticoagulant, fibrinolytic, and control serine proteases. No measurable inhibition was observed for Factor Xa (FXa), Factor XIa (FXIa), Factor XIIa (FXIIa), plasma kallikrein, activated protein C (APC), plasmin, or trypsin at concentrations up to 10 µM. Similarly, no inhibition of Factor VIII (FVIII) or Factor IX (FIX) activity was detected in chromogenic assays (Table 1, Supplementary Figure S1). In contrast, AYA1809002 potently inhibited thrombin-dependent fibrin formation with a functional IC_50_ of 25.7 nM, demonstrating a high degree of target selectivity spanning more than two orders of magnitude.

**Figure 5:**
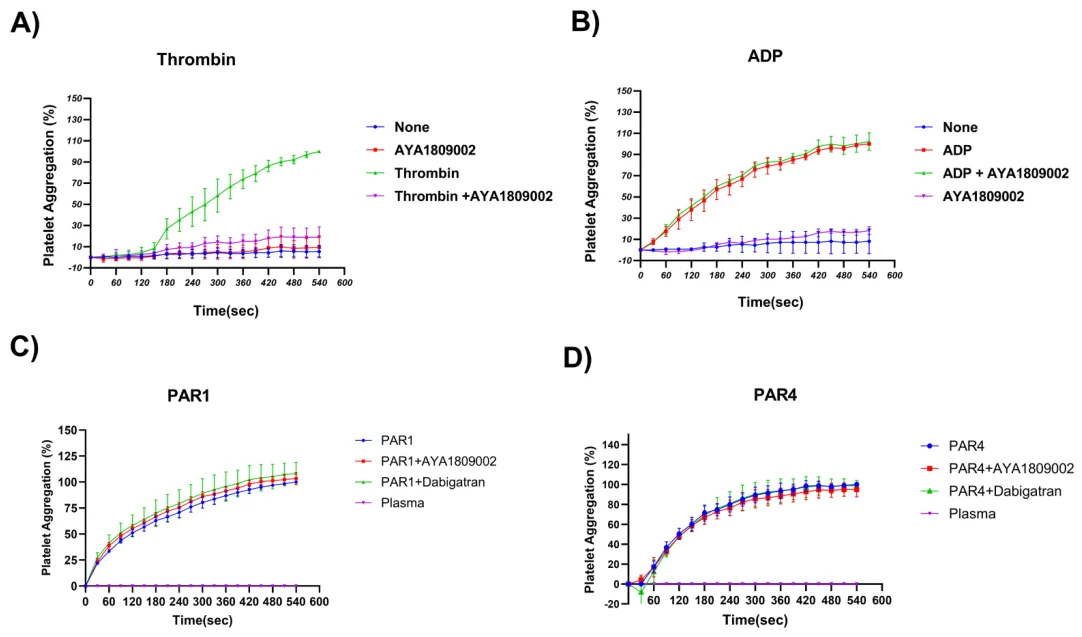
AYA1809002 selectively inhibits thrombin-dependent platelet aggregation. Platelet aggregation in human platelet-rich plasma was induced by (**A**) thrombin, (**B**) ADP, (**C**) PAR1-activating peptide, or (**D**) PAR4-activating peptide in the presence or absence of AYA1809002 (1 µM). All data are representative of at least two independent experiments. Error bars represented as mean ± SD of the independent experiment in triplicate.

Functional selectivity was further assessed using platelet aggregation assays in human platelet-rich plasma. AYA1809002 (1 µM) markedly inhibited thrombin-induced platelet aggregation but had no effect on platelet activation induced by ADP (Figure 5A,B). Additional studies demonstrated no inhibition of aggregation induced by PAR1-activating peptide, PAR4-activating peptide [28,29] (Figure 5C,D), collagen, or the thromboxane A_2_ receptor agonist U46619 (Supplementary Figure S2).

Together, these findings demonstrate that AYA1809002 selectively inhibits thrombin-dependent platelet aggregation under the tested conditions while preserving platelet responses to thrombin-independent agonists, supporting a mechanism mediated by inhibition of thrombin signaling rather than nonspecific suppression of platelet function. Collectively, these results demonstrate that AYA1809002 exhibits a highly selective thrombin-centered anticoagulant profile characterized by potent inhibition of thrombin-dependent coagulation.

### 2.4. AYA1809002 Preserves Thrombomodulin-Mediated Protein C Activation

**Figure 6:**
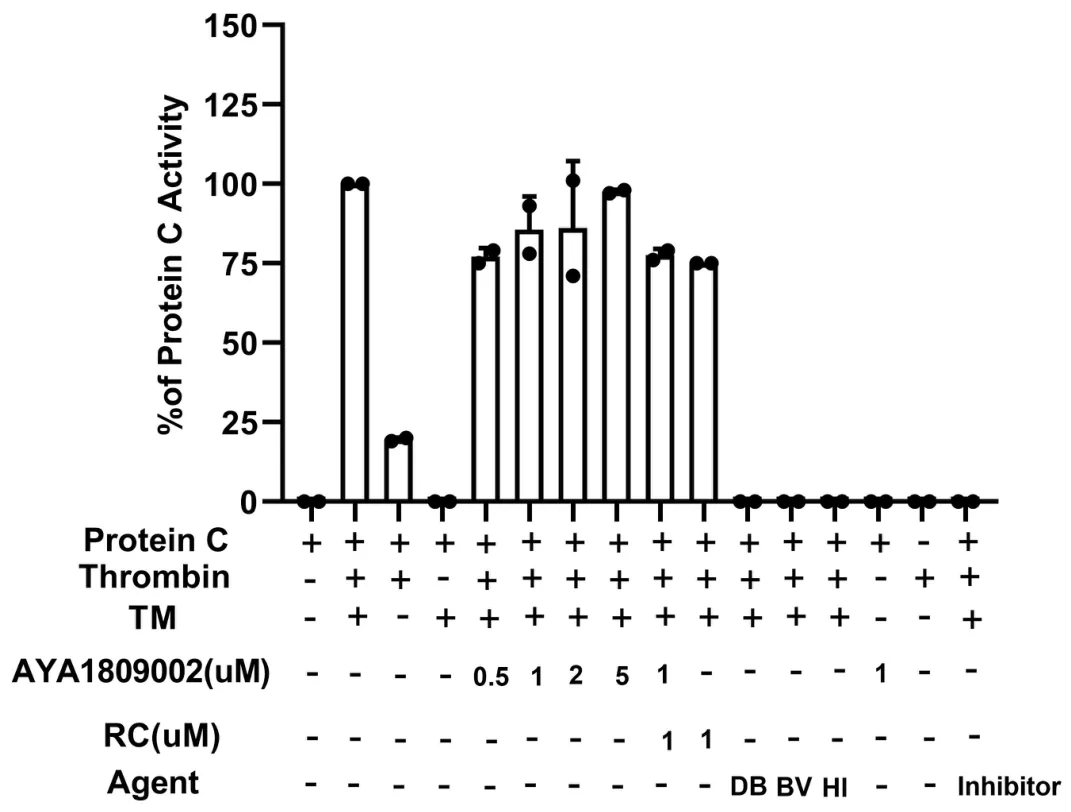
AYA1809002 preserves thrombomodulin-mediated protein C activation. Purified human protein C (PrC) was incubated with human α-thrombin and thrombomodulin (TM) for 30 min at 37 °C in the absence or presence of AYA1809002 (0.5–5 µM). Thrombin was subsequently quenched with hirudin, and generated APC activity was measured using Spectrozyme^®^ PCa. APC generation in the PrC + thrombin + TM condition was defined as 100%. Reverse-complement oligonucleotide (RC; 1:1 molar ratio to AYA1809002) was evaluated for reversal. Dabigatran (DB; 1 µM), bivalirudin (BV; 2 µM), and hirudin (HI; 50 nM) were included as comparator thrombin inhibitors, and recombinant human serpin was used as an APC inhibition control. Data are expressed as percentage of the PrC + thrombin + TM control. Data represent mean ± SD from two independent experiments, each performed in triplicate.

Because thrombin bound to thrombomodulin activates protein C, we investigated whether inhibition of thrombin through Exosite I affects this endogenous anticoagulant pathway. As expected, protein C alone or protein C incubated with thrombomodulin alone generated negligible APC activity, whereas thrombin alone produced only limited activation. In contrast, the combination of thrombin and thrombomodulin efficiently generated APC and was defined as 100% activity. Addition of AYA1809002 at concentrations ranging from 0.5 to 5 µM preserved APC generation, maintaining 86–109% of control activity with no concentration-dependent inhibition. Similarly, addition of the reverse-complement (RC) oligonucleotide had no measurable effect on APC generation. In contrast, the active-site thrombin inhibitors dabigatran, bivalirudin, and hirudin nearly completely abolished APC generation under identical experimental conditions. These findings demonstrate that selective inhibition of thrombin through Exosite I preserves thrombomodulin-dependent protein C activation, distinguishing AYA1809002 mechanistically from conventional active-site thrombin inhibitors (Figure 6).

### 2.5. Reverse-Complement Oligonucleotide Provides Rapid and Durable Reversal of AYA1809002 Activity

**Figure 7:**
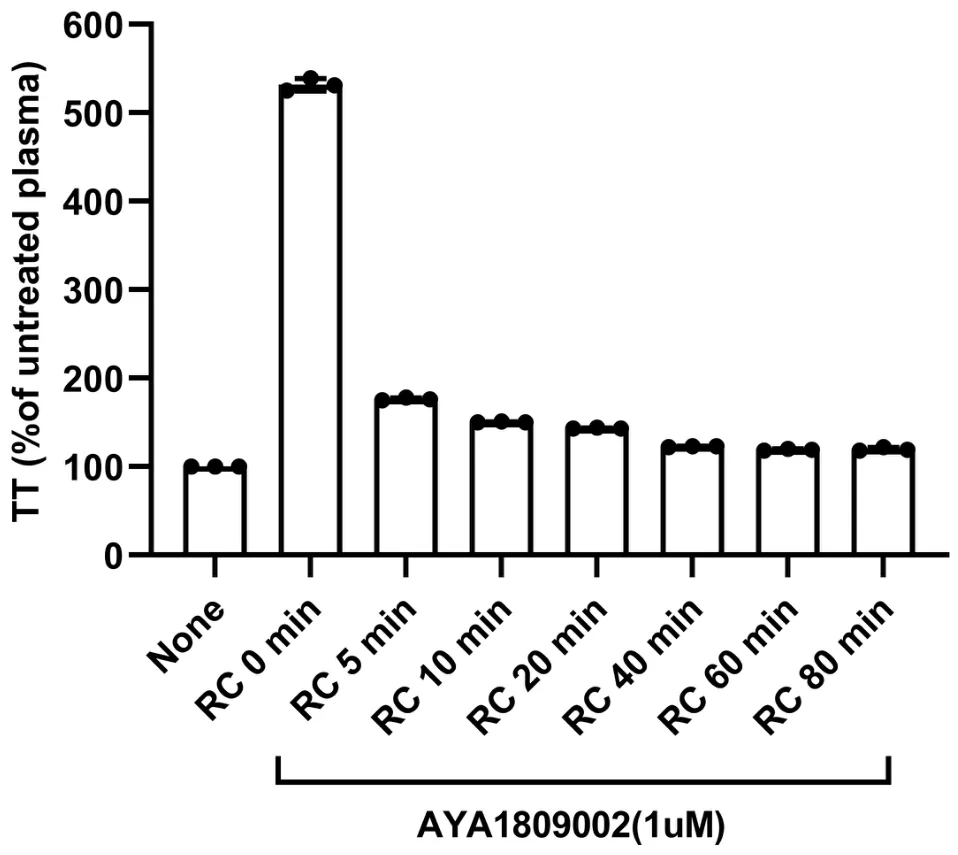
Rapid reversal of AYA1809002-induced thrombin time prolongation by a reverse-complement (RC) oligonucleotide. Pooled citrated human plasma was incubated with AYA1809002 (1 µM) for 1 h, followed by addition of reverse-complement (RC) at a 1:1 molar ratio. TT was measured at the indicated times after RC addition and expressed relative to untreated plasma. Data represent mean ± SD from three independent experiments, each performed in triplicate.

The reversibility of AYA1809002 anticoagulant activity was first evaluated using thrombin time (TT) as a functional readout. Incubation of pooled citrated human plasma with AYA1809002 (1 µM) produced marked prolongation of TT relative to untreated plasma. Addition of a reverse-complement (RC) oligonucleotide at a 1:1 molar ratio resulted in rapid reduction of TT prolongation, with substantial reversal observed within 5 min and progressive recovery toward baseline values over the subsequent 80 min (Figure 7).

**Figure 8:**
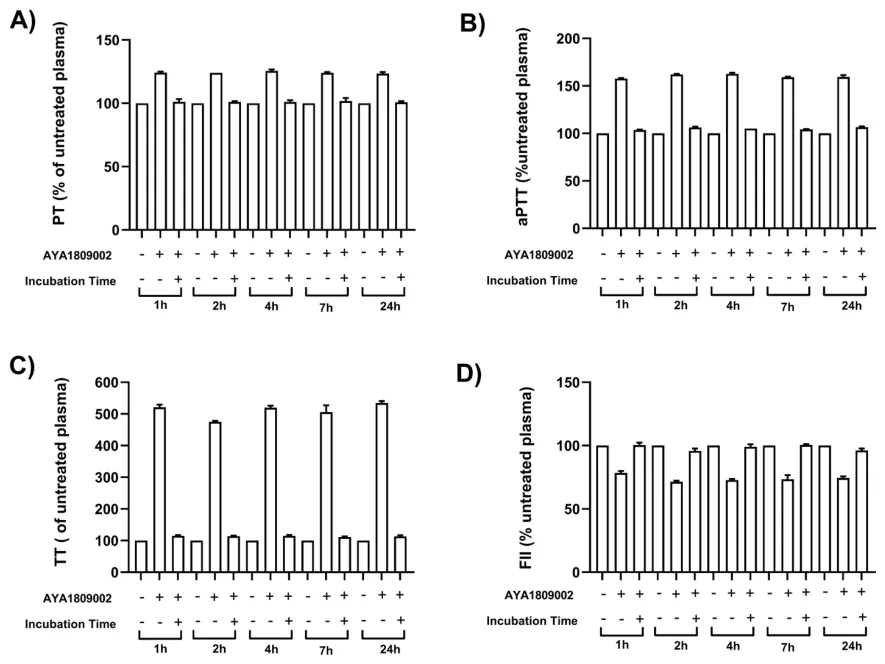
Reversal of AYA1809002 anticoagulant activity after prolonged plasma exposure. Pooled citrated human plasma was incubated with AYA1809002 (1 µM) for 1–24 h, followed by RC (1:1 molar ratio). After 1 h, (**A**) PT, (**B**) aPTT, (**C**) TT, and (**D**) Factor II activity were measured and normalized to the corresponding untreated plasma control at each time point. Untreated control values are provided in Supplementary Table S1. Data represent mean ± SD from five independent experiments, each performed in triplicate.

To determine whether reversal remained effective following prolonged plasma exposure, pooled plasma was incubated with AYA1809002 (1 µM) for 1, 2, 4, 7, or 24 h prior to RC addition. To account for potential time-dependent changes in plasma coagulation properties during prolonged incubation, all measurements were normalized to the corresponding untreated plasma control at each incubation time point. Untreated plasma controls exhibited only minimal variation over the 24-h study period, supporting the validity of this normalization approach (Supplementary Table S1). In the absence of antidote, AYA1809002 consistently prolonged PT, aPTT, and TT and reduced Factor II activity throughout the incubation period. Addition of the RC antidote restored PT (Figure 8A), aPTT (Figure 8B), TT (Figure 8C), and Factor II activity (Figure 8D) toward baseline values at all time points examined.

Collectively, these findings demonstrate that AYA1809002 anticoagulant activity can be rapidly and effectively neutralized by a sequence-specific reverse-complement oligonucleotide and that this reversibility is maintained after prolonged exposure in plasma.

### 2.6. AYA1809002 Exhibits Favorable Stability Characteristics

**Figure 9:**
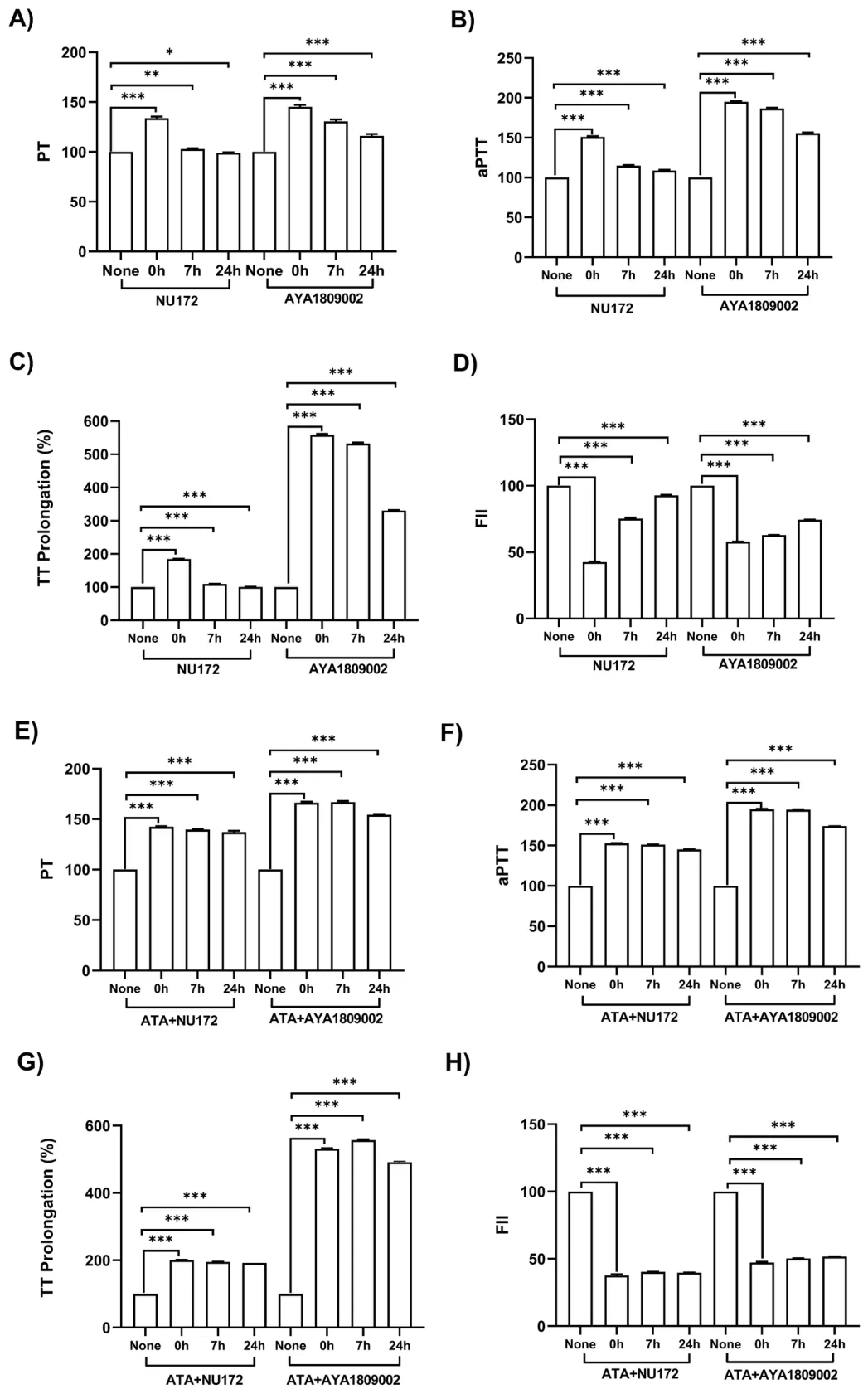
Functional serum stability of AYA1809002 compared with NU172. AYA1809002 and NU172 were incubated in human serum at 37 °C for the indicated times in the absence or presence of aurintricarboxylic acid (ATA). Residual anticoagulant activity was assessed after transfer to pooled human plasma. (**A**) PT, (**B**) aPTT, (**C**) TT, and (**D**) FII activity represent anticoagulant activity in the absence of ATA. (**E**) PT, (**F**) aPTT, (**G**) TT, and (**H**) FII activity represent anticoagulant activity in the presence of ATA. Data are representative of at least three independent experiments. Error bars represent mean ± SD of triplicate measurements. Statistical comparisons versus the untreated control (None) were performed using two-sided paired Student’s *t*-tests with Holm adjustment for multiple comparisons (Supplementary Table S4) * indicated *p* < 0.05. ** indicated *p* < 0.01. *** indicated *p*-value < 0.001.

Functional stability of AYA1809002 was evaluated under biological and physicochemical conditions relevant to therapeutic development. Functional stability in human serum was assessed using thrombin time (TT) as a measure of residual anticoagulant activity and compared with the clinically evaluated thrombin aptamer NU172. Following incubation in human serum at 37 °C, AYA1809002 retained substantial anticoagulant activity for up to 24 h, whereas NU172 exhibited a marked loss of activity over the same period (Figure 9, Table 2, Supplementary Table S4). AYA1809002 maintained greater thrombin-inhibitory activity than NU172 in the absence of the nuclease inhibitor aurintricarboxylic acid (ATA), demonstrating greater functional persistence following serum exposure. Preservation of anticoagulant activity in the presence of ATA is consistent with nuclease-mediated degradation contributing to the loss of functional activity during serum incubation; however, oligonucleotide integrity was not directly quantified in this experiment.

As part of a broader characterization of the biochemical robustness of AYA1809002, stability was also evaluated under simulated gastrointestinal stress conditions, including acidic and enzymatic exposure. These exploratory experiments were intended to assess susceptibility of the unmodified DNA aptamer to degradation under distinct biological stress conditions rather than to evaluate oral delivery or bioavailability (Supplementary Figure S3). These findings indicate that AYA1809002 is resistant to several physiologically relevant digestive conditions but remains susceptible to nuclease degradation. Additional physicochemical stability studies were performed under accelerated stress conditions, including elevated temperature, oxidative challenge [30,31], light exposure, and pH variation. AYA1809002 retained anticoagulant activity across a broad range of tested conditions, demonstrating favorable stability during environmental and formulation-related stress [32,33] (Supplementary Figures S4–S5).

AYA1809002 retained stability under the accelerated thermal stress conditions evaluated at 50 °C and 60 °C. Q10-based calculations were additionally performed as an exploratory means of estimating the potential relationship between accelerated and ambient-temperature stability. These calculations generated theoretical equivalent durations at 25 °C; however, because the projections have not been validated by real-time stability testing, they should be regarded as preliminary estimates rather than experimentally established shelf-life values [34,35,36] (Table 3).

Collectively, these findings demonstrate that AYA1809002 possesses favorable biological and physicochemical stability characteristics, exhibits improved functional stability relative to NU172, and maintains activity under conditions relevant to pharmaceutical development and storage.

### 2.7. Translational Development of AYA1809002

**Figure 10:**
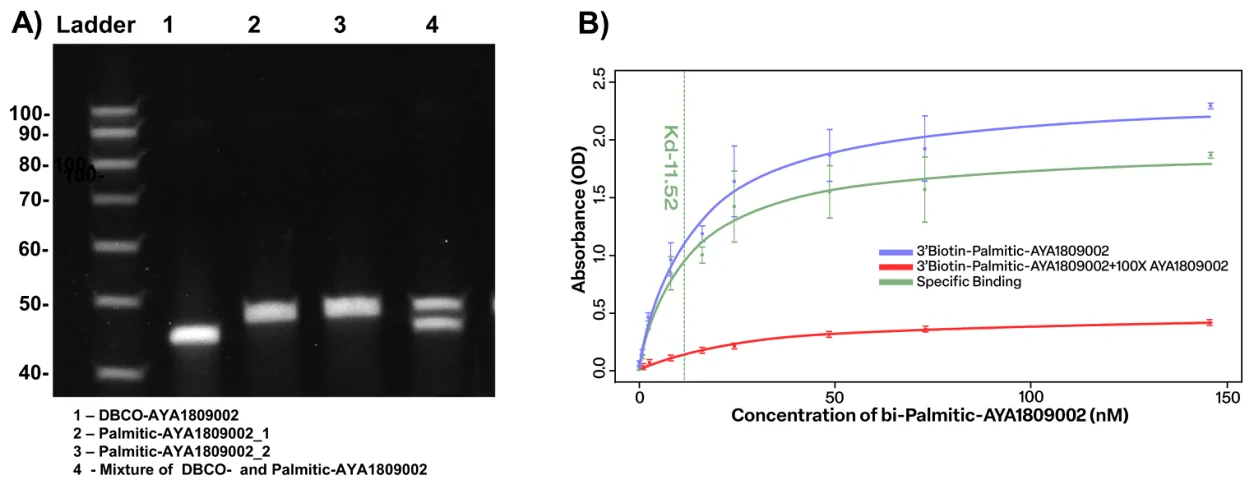
Palmitic acid conjugation and thrombin binding of Palmitic-AYA1809002. (**A**) Denaturing TBE-urea PAGE of DBCO-AYA1809002 (lane 1), two Palmitic-AYA1809002 preparations (lanes 2–3), and a mixture of unconjugated and conjugated aptamer (lane 4). (**B**) Binding of Palmitic-AYA1809002 to immobilized human α-thrombin measured by competitive enzyme-linked binding assay. Nonlinear regression yielded an apparent K_*D*_ of 11.5 nM. RMSE values for $3^{\prime }$ Biotin-Palmitic-AYA1809002 binding, binding in the presence of 100-fold excess AYA1809002, and specific binding were 0.06895, 0.01455, and 0.06656, respectively. Data represent mean ± SD of duplicate measurements.

To enhance the translational potential of AYA1809002, AYA1809002 was modified at the $5^{\prime }$ terminus with palmitic acid as a development strategy intended to increase systemic exposure, based on the established use of fatty-acid conjugation to promote serum-protein association and reduce renal clearance of oligonucleotide and peptide therapeutics. Successful conjugation was confirmed by denaturing PAGE, which demonstrated the expected reduction in electrophoretic mobility relative to the unconjugated precursor (Figure 10A). Binding affinity was evaluated using a competitive enzyme-linked binding assay, which demonstrated that Palmitic-AYA1809002 retained high-affinity thrombin recognition with an estimated K_*D*_ of approximately 11.5 nM (Figure 10B). These results indicate that lipid modification did not substantially impair thrombin binding.

**Figure 11:**
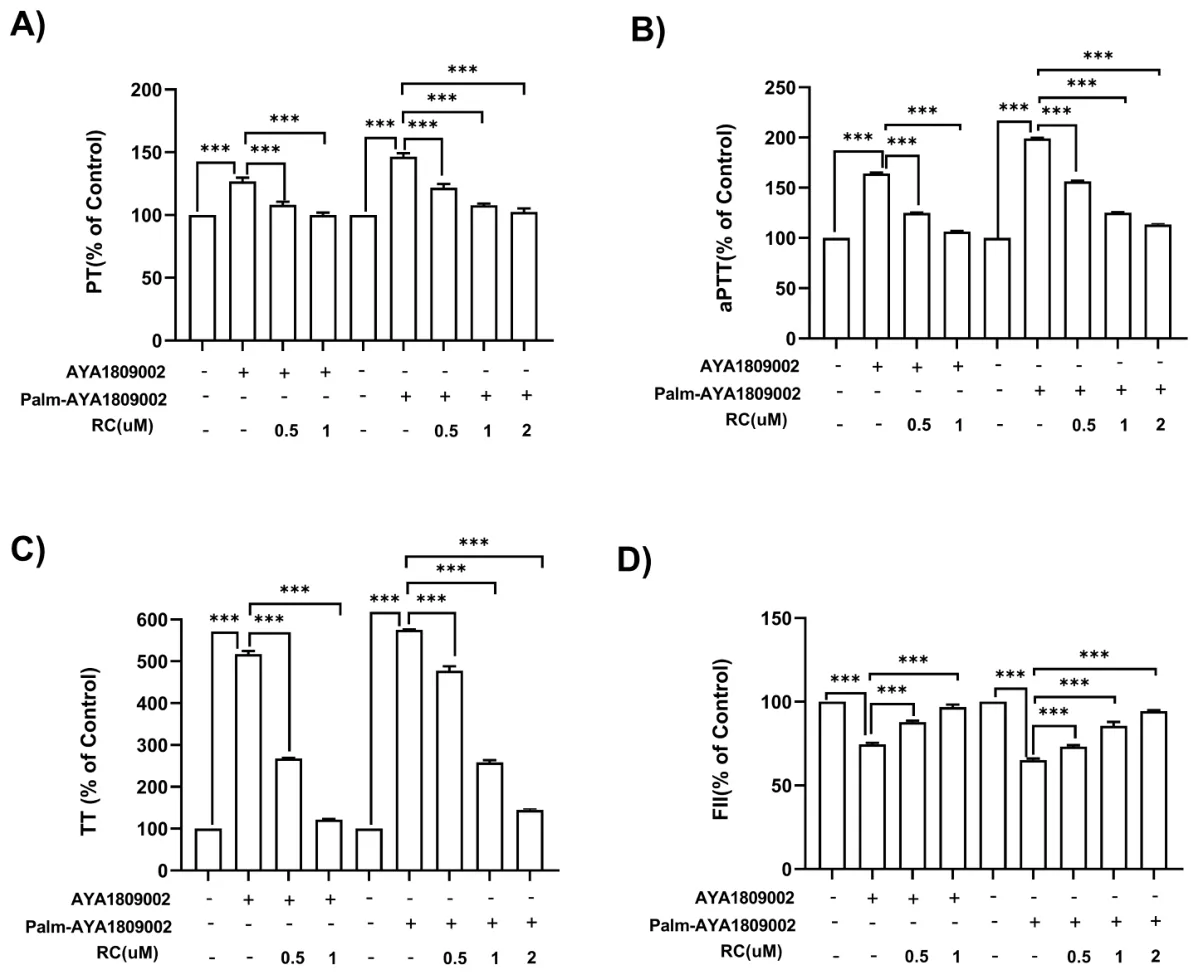
Palmitic -AYA1809002 retains anticoagulant activity and complementary-oligonucleotide reversibility. Pooled citrated human plasma was treated with Palmitic-AYA1809002 (1 µM) alone or followed by RC at the indicated RC:aptamer molar ratios. (**A**) PT, (**B**) aPTT, (**C**) TT, and (**D**) Factor II activity are expressed relative to untreated plasma. Data represent mean ± SD from three independent experiments, each performed in triplicate. Statistical comparisons were performed using two-sided paired Student’s *t*-tests with Holm adjustment for multiple comparisons (Supplementary Table S5). *** indicated *p*-value < 0.001.

The functional consequences of palmitic acid conjugation were evaluated in pooled citrated human plasma. Palmitic-AYA1809002 retained anticoagulant activity, producing prolongation of PT, aPTT, and TT comparable to that observed with the parent aptamer. Addition of a reverse-complement (RC) resulted in dose-dependent normalization of coagulation parameters and restoration of Factor II activity, demonstrating preservation of sequence-specific reversibility following lipid conjugation (Figure 11, Supplementary Table S5).

**Figure 12:**
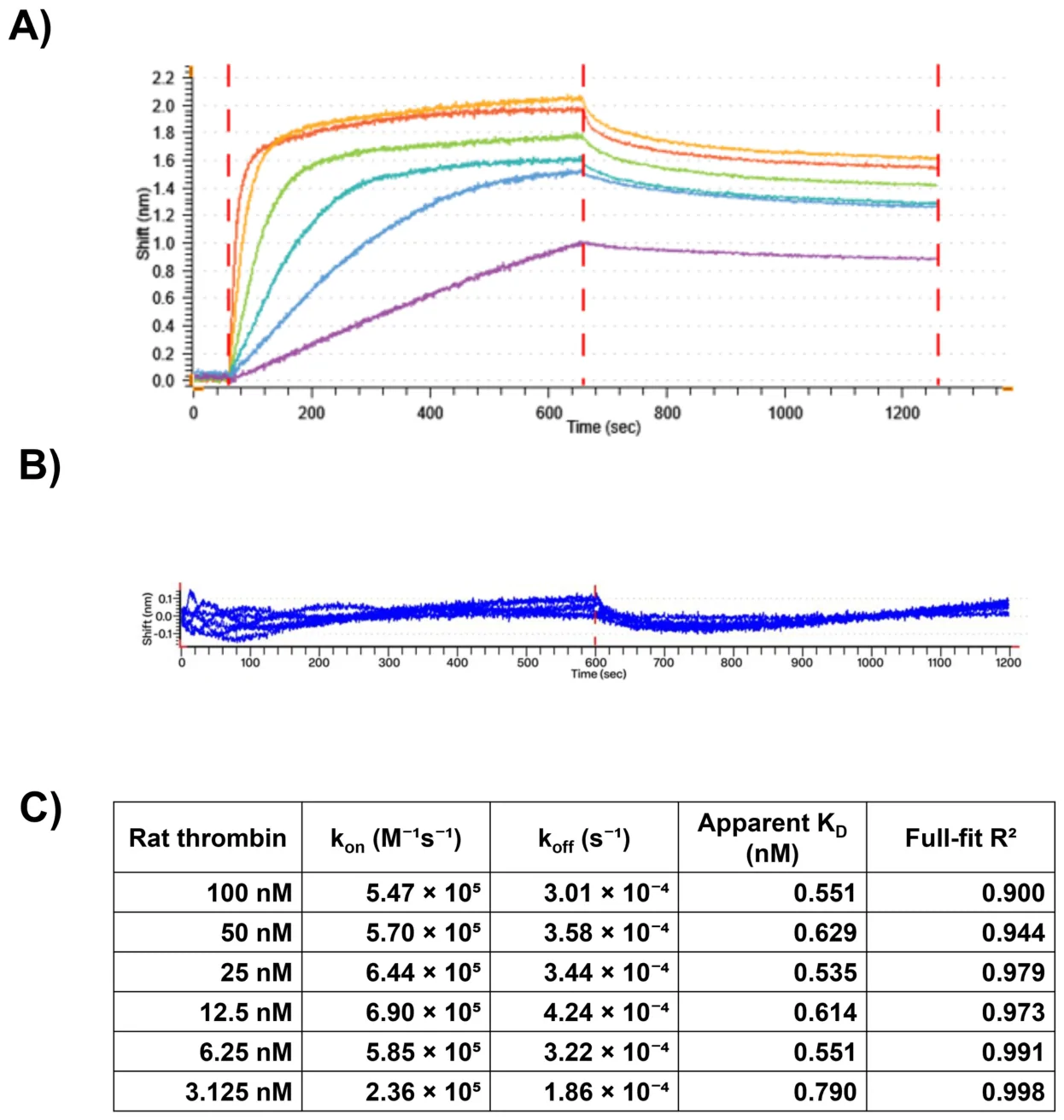
Biolayer interferometric analysis of AYA1809002 binding to rat thrombin. (**A**) BLI sensorgrams and fitted curves for rat thrombin at 3.125–100 nM. Biotinylated AYA1809002 was immobilized on streptavidin biosensors, and association and dissociation were monitored for 600 s each. Sensorgrams were reference-subtracted and fitted using a 1:1 Langmuir interaction model in GatorOne software. (**B**) Residual plots corresponding to the fitted sensorgrams. (**C**) Apparent kinetic parameters and full-fit R^2^ values obtained from the kinetic analysis.

AYA1809002 binding to rat thrombin was evaluated by BLI to assess cross-species conservation of thrombin recognition. AYA1809002 exhibited concentration-dependent binding to rat thrombin over the tested concentration range (3.125–100 nM), characterized by rapid association and slow dissociation (Figure 12A). Sensorgrams were fitted using a 1:1 Langmuir interaction model, yielding apparent K_*D*_ values of 0.535–0.790 nM. The corresponding k_*on*_ and k_*off*_ values ranged from 2.36 × 10^5^ to 6.90 × 10^5^ M^−1^s^−1^ and 1.86 × 10^−4^ to 4.24 × 10^−4^ s^−1^, respectively. Full-fit R^2^ values ranged from 0.900 to 0.998, and the corresponding residual curves are shown in Figure 12B. Kinetic analysis using a 1:1 Langmuir interaction model yielded an average equilibrium dissociation constant (K_*D*_) of approximately 6.1 × 10^−10^ M, an association rate constant (k_*on*_) of approximately 5.5 × 10^5^ M^−1^s^−1^, and a dissociation rate constant (k_*off*_) of approximately 3.2 × 10^−4^ s^−1^ (Figure 12C), corresponding to a thrombin–aptamer complex half-life of approximately 36 min, consistent with formation of a stable thrombin-bound complex. These results demonstrate high-affinity binding of AYA1809002 to rat thrombin and support cross-species conservation of its thrombin-binding activity.

**Figure 13:**
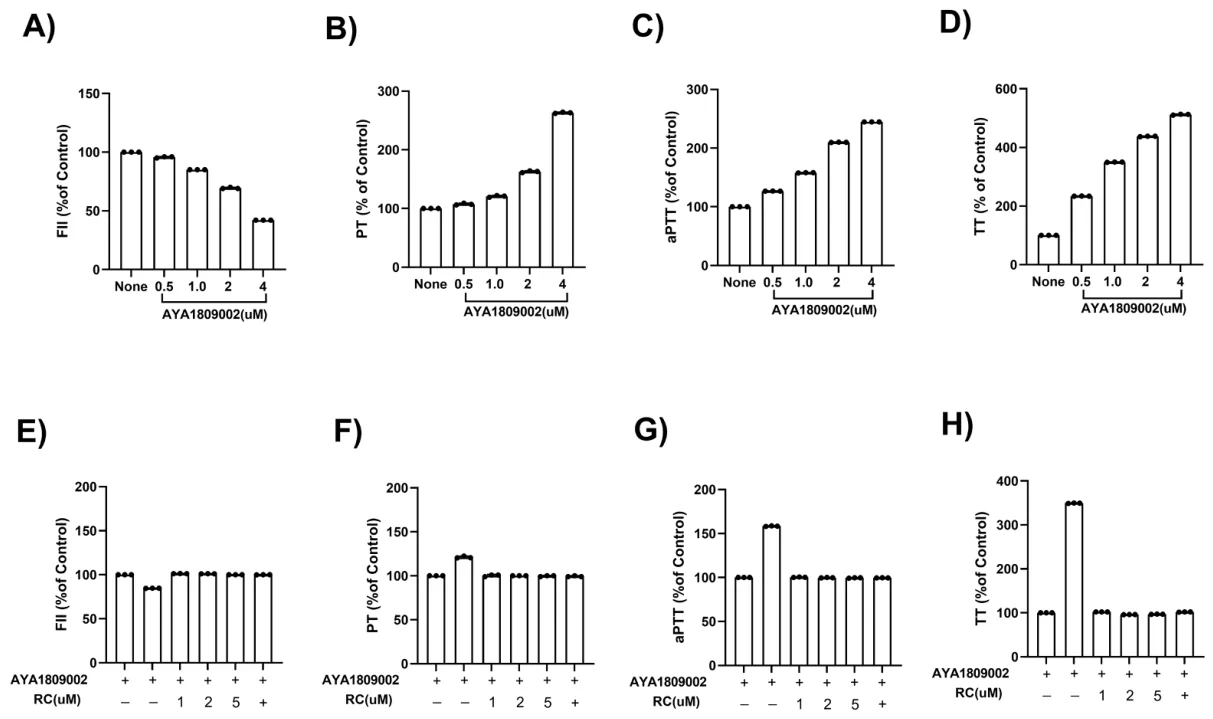
Anticoagulant activity and reversibility of AYA1809002 in rat plasma. (**A**–**D**) Concentration-dependent effects of AYA1809002 on Factor II activity, PT, aPTT, and TT in citrated rat plasma. (**E**–**H**) Reversal following treatment with AYA1809002 (1 µM) and RC at the indicated RC: aptamer molar ratios. RC alone served as a control. Results are expressed relative to untreated plasma. Data represent mean ± SD from three independent experiments, each performed in triplicate.

Consistent with the binding studies, AYA1809002 produced dose-dependent anticoagulant activity in rat plasma and retained full reversibility following addition of the RC antidote (Figure 13). Together, these findings demonstrate that AYA1809002 retains activity and programmable reversibility following lipid conjugation and maintains high-affinity thrombin recognition and anticoagulant activity across species, supporting its continued preclinical development.

## 3. Discussion

Thrombin remains one of the most important therapeutic targets in anticoagulation because of its central role in fibrin formation, coagulation amplification, and platelet activation [13,24,25]. However, currently approved anticoagulants that target thrombin typically function through catalytic active-site inhibition, which suppresses essentially all thrombin proteolytic activity. As a result, both procoagulant and anticoagulant functions of thrombin are inhibited, limiting the ability to selectively modulate thrombin biology. These limitations have stimulated interest in alternative strategies capable of controlling specific thrombin functions while preserving other physiologically important activities [2,7,24,37].

In the present study, we provide a comprehensive mechanistic and translational characterization of AYA1809002, a DNA aptamer that selectively targets thrombin Exosite I. Our results demonstrate that AYA1809002 functions as a substrate-selective thrombin inhibitor with programmable reversibility, favorable stability characteristics, and retained activity following lipid conjugation. Collectively, these findings position AYA1809002 as a promising preclinical aptamer for controllable anticoagulant therapy and provide a strong foundation for further preclinical development.

### 3.1. Mechanism of Action: Exosite I Engagement and Substrate Selectivity

Multiple orthogonal experimental approaches demonstrate that AYA1809002 interacts with thrombin primarily through Exosite I, the fibrinogen-recognition surface of thrombin. Competitive binding assays showed strong displacement by canonical Exosite I ligands such as hirudin and the reference aptamer NU172, whereas inhibitors targeting the catalytic active site such as dabigatran produced minimal competition (Figure 2A).

Interestingly, fibrinogen produced little competition in the competitive enzyme-linked binding assay despite the strong functional inhibition of fibrinogen cleavage observed in thrombin time studies [25,38]. The absence of measurable fibrinogen-mediated displacement in the competition assay should not be interpreted as evidence that fibrinogen and AYA1809002 bind completely distinct thrombin surfaces. Exosite I is an extended macromolecular recognition interface, and different ligands can engage overlapping but nonidentical subsets of residues within this region. Furthermore, the immobilized competition format measures the ability of a soluble ligand to displace surface-associated aptamer binding under defined assay conditions and does not constitute a reciprocal equilibrium competition analysis. Accordingly, Exosite I localization of AYA1809002 is supported primarily by strong competition with NU172 and hirudin and by the marked loss of binding to γ-thrombin. Functional interference with fibrinogen recognition is independently supported by the potent inhibition of fibrin formation despite preservation of thrombin catalytic activity toward a small peptide substrate (Figure 2B).

Collectively, these findings provide direct experimental evidence that AYA1809002 recognition depends on an intact Exosite I surface and further exclude the thrombin catalytic site as the primary binding locus.

The functional consequences of Exosite I occupancy are particularly informative. AYA1809002 did not inhibit thrombin-mediated cleavage of a small fluorogenic peptide substrate at concentrations up to 10 µM (Figure 3A), indicating that the catalytic active site remains fully accessible. In marked contrast, the aptamer potently inhibited fibrin formation in thrombin time assays, with a functional IC_50_ of approximately 26 nM and a calculated K_*i*_ of approximately 10.6 nM (Figure 3B–D). This dissociation between preserved catalytic turnover toward small substrates and selective inhibition of macromolecular substrate cleavage is the hallmark of Exosite-mediated thrombin inhibition, a mechanism previously described for hirudin derivatives and certain RNA aptamers [12,19,37]. By contrast, dabigatran suppresses cleavage of both small peptides and physiological substrates indiscriminately [8,9]. The selective inhibition profile observed for AYA1809002 therefore represents a mechanistically distinct mode of thrombin modulation with potential safety advantages.

### 3.2. Thrombin-Centered Anticoagulant Profile and Biochemical Selectivity

Coagulation assays in human plasma demonstrated that AYA1809002 produces a thrombin-centered anticoagulant profile. The aptamer markedly prolonged thrombin time while producing only moderate prolongation of activated partial thromboplastin time, consistent with selective inhibition of thrombin-mediated fibrin formation. This profile differed from that of NU172 and was mechanistically distinct from dabigatran, which completely suppressed clot formation under the same assay conditions. Direct biochemical profiling further demonstrated a high degree of target selectivity. AYA1809002 potently inhibited thrombin, whereas no measurable inhibitory activity was observed against Factor Xa, Factor XIa, Factor XIIa, plasma kallikrein, activated protein C, plasmin, trypsin, Factor VIII, or Factor IX at concentrations up to 10 µM. The absence of measurable activity against coagulation, anticoagulant, and fibrinolytic proteases further supports the conclusion that the anticoagulant effects of AYA1809002 arise from selective thrombin modulation rather than off-target protease inhibition.

An important mechanistic finding of the present study is that AYA1809002 also preserved thrombomodulin-dependent Protein C activation despite potent inhibition of thrombin-mediated fibrin formation. Thrombomodulin binding alters thrombin substrate specificity and promotes conversion of Protein C to activated Protein C (APC), thereby contributing to endogenous anticoagulant regulation. Although AYA1809002 targets the Exosite I region of thrombin and strongly inhibits fibrinogen cleavage, it did not suppress APC generation over the concentration range tested. In contrast, dabigatran, bivalirudin, and hirudin markedly reduced APC generation, consistent with broader inhibition of thrombin proteolytic activity. Together with the absence of direct APC inhibition, these findings support a mechanism in which AYA1809002 preferentially suppresses procoagulant thrombin functions while preserving thrombomodulin-dependent Protein C activation. This functional selectivity may represent an important mechanistic feature of Exosite I-targeted thrombin inhibition and warrants further evaluation under physiologic and in vivo conditions.

### 3.3. Selective Effects on Platelet Activation

Thrombin contributes to thrombus formation not only through fibrin generation but also through activation of platelets via PAR1 and PAR4 [7,10]. Consistent with its thrombin-centered mechanism of action, AYA1809002 effectively inhibited thrombin-induced platelet aggregation while preserving responses to ADP, collagen, U46619, and direct PAR1 and PAR4 activation. The absence of inhibition following PAR1-activation peptide or PAR4-activation peptide stimulation indicates that AYA1809002 acts upstream of receptor activation by preventing thrombin-mediated receptor cleavage rather than interfering with downstream platelet signaling pathways.

The platelet aggregation experiments in the present study were designed primarily to assess mechanistic selectivity rather than concentration-dependent potency. AYA1809002 inhibited thrombin-induced aggregation while preserving responses to thrombin-independent platelet agonists, supporting a thrombin-dependent mechanism rather than nonspecific platelet inhibition. A limitation of the present analysis is that a full concentration-response study was not performed; therefore, a platelet-specific IC_50_ cannot be derived from these data. Concentration-dependent studies in platelet-rich plasma would provide additional quantitative characterization of the antiplatelet effects of AYA1809002.

### 3.4. Programmable Reversibility: A Pharmacological Advantage

A unique advantage of aptamer-based therapeutics is the ability to neutralize their activity using reverse-complement (RC) oligonucleotides that disrupt aptamer structure through Watson–Crick hybridization [15,16,22]. AYA1809002 demonstrated rapid and robust reversibility across multiple coagulation parameters. Addition of a reverse- complement oligonucleotide rapidly restored thrombin time toward baseline within minutes (Figure 7), and anticoagulant activity remained fully reversible even after prolonged plasma exposure (Figure 8). Such programmable anticoagulant control represents a major pharmacological advantage compared with conventional anticoagulants, where reversal often requires administration of specialized biologic agents such as idarucizumab or andexanet alfa [3,4]. Beyond pharmacological considerations, the use of a short complementary DNA oligonucleotide as the reversal agent offers potential practical and economic advantages over antibody-based reversal strategies, which require intravenous administration in monitored clinical settings and carry substantial per-dose costs. The simplicity and scalability of oligonucleotide synthesis may therefore support more accessible reversal options across a broader range of clinical environments.

### 3.5. Improved Stability Relative to NU172

The enhanced functional persistence of AYA1809002 relative to NU172 may be related to sequence-dependent differences in oligonucleotide folding and structural organization. In our previous study, computational secondary-structure analysis of AYA1809002 using RNAfold predicted a hairpin architecture containing unpaired loop regions [21]. AYA1809002 is also G-rich, suggesting the potential formation of higher-order G-quadruplex structures [21]. In comparison, NU172 has been structurally characterized as a bimodular DNA aptamer comprising an antiparallel G-quadruplex connected to a duplex domain [39]. NU172 was selected as a clinically relevant comparator because it is an unmodified thrombin-targeting DNA aptamer that progressed to clinical evaluation, including a Phase II study [39]. Importantly, both AYA1809002 and NU172 evaluated in the present study are unmodified DNA oligonucleotides; therefore, the greater functional persistence observed for AYA1809002 cannot be attributed to nuclease-resistant chemical modifications. Rather, the difference may reflect intrinsic sequence- and structure-dependent properties of the two aptamers. However, because the secondary structure of AYA1809002 was computationally predicted rather than experimentally determined, the molecular basis for its enhanced functional persistence remains to be established by direct structural and nuclease-degradation studies.

### 3.6. Stability Profile of AYA1809002

The accelerated thermal stability findings provide preliminary information regarding the robustness of AYA1809002 under elevated-temperature stress. Although Q10-based extrapolation can provide an exploratory estimate of temperature-dependent stability, such calculations do not substitute for real-time stability studies conducted under defined storage conditions. Accordingly, the Q10-derived values reported here should be interpreted only as preliminary projections and not as validated shelf-life estimates. Establishment of a formal shelf life for AYA1809002 will require longitudinal stability studies incorporating direct assessment of oligonucleotide integrity, purity, and biological activity under the intended formulation and storage conditions.

The gastrointestinal stability experiments were included as exploratory biochemical stress tests complementing the serum and nuclease stability studies and were not intended to imply oral administration or bioavailability of AYA1809002.

### 3.7. Lipid Conjugation and Cross-Species Activity

To address the rapid renal clearance characteristic of unmodified oligonucleotides, AYA1809002 was conjugated to palmitic acid at the $5^{\prime }$ terminus via copper-free click chemistry, a strategy previously explored to promote serum-protein association and potentially reduce renal clearance [16,40,41,42]. Palmitic-AYA1809002 retained high-affinity thrombin binding (K_*D*_ ≈ 11.5 nM), as determined by a competitive enzyme-linked binding assay, and preserved anticoagulant activity in human plasma (Figure 10 and Figure 11). Importantly, sequence-specific reversal by the reverse-complement (RC) oligonucleotide was also fully maintained, indicating that palmitic acid conjugation does not compromise the thrombin-binding, anticoagulant, or programmable reversal properties of the parent aptamer. Although fatty-acid conjugation may promote serum-protein association and thereby potentially reduce renal clearance, direct albumin binding and pharmacokinetic parameters, including systemic exposure and half-life, were not evaluated in the present study. In addition, conjugate identity and purity were confirmed by denaturing PAGE mobility shift rather than by mass spectrometry; definitive analytical characterization of Palmitic-AYA1809002 by mass spectrometry was not performed in the present study and represents an important component of future developability and chemistry, manufacturing, and controls (CMC) characterization. Thus, these findings demonstrate that palmitic acid conjugation is compatible with the key pharmacologic properties of AYA1809002, while its potential to improve systemic exposure and prolong pharmacokinetic half-life remains to be established in dedicated protein-binding and pharmacokinetic studies. Palmitic acid conjugation was chosen over more conventional half-life-extension strategies such as PEGylation in part because PEGylated therapeutics, including a PEGylated anticoagulant RNA aptamer (pegnivacogin), have been associated with severe, PEG-directed hypersensitivity reactions in clinical trials attributable to pre-existing anti-PEG antibodies [43]. Fatty-acid conjugation avoids introduction of a PEG moiety and its associated immunogenicity risk while still promoting serum-protein association to reduce renal clearance, supporting the pharmaceutical rationale for this conjugation strategy. It is also theoretically possible that albumin association mediated by the palmitic acid moiety could sterically hinder the interaction of Palmitic-AYA1809002 with thrombin Exosite I or interfere with hybridization of the RC antidote to the aptamer, which could in principle modulate the pharmacological activity or reversibility of the lipid-conjugated aptamer in vivo. This possibility was not directly assessed in the present in vitro system and should be evaluated together with albumin-binding and pharmacokinetic studies.

Cross-species activity was evaluated to determine whether the thrombin-binding and anticoagulant properties of AYA1809002 are preserved in the rat system. Biolayer interferometry showed that AYA1809002 binds rat thrombin with subnanomolar affinity (K_*D*_ ≈ 0.6 nM), characterized by rapid association and slow dissociation kinetics. Functional studies further demonstrated dose-dependent anticoagulant activity in rat plasma, and these effects remained reversible following addition of the reverse-complement (RC) oligonucleotide (Figure 12 and Figure 13). Together, these findings demonstrate cross-species preservation of the key biochemical and functional properties of AYA1809002, including high-affinity thrombin binding, anticoagulant activity, and programmable reversibility. However, these in vitro findings do not establish in vivo pharmacological activity, which will require dedicated pharmacokinetic, pharmacodynamic, thrombosis, bleeding, and safety studies.

### 3.8. Aptamer Comparison

AYA1809002 can also be considered in the context of established and emerging thrombin-targeting aptamers developed as anticoagulant or antithrombotic agents. The canonical 15-nucleotide thrombin-binding aptamer HD1/TBA (ARC183) targets Exosite I and inhibits thrombin interactions with fibrinogen [44]. NU172, a 26-nucleotide unmodified DNA aptamer also targeting Exosite I, subsequently progressed to clinical evaluation, including a Phase II study [39]. RE31 represents another Exosite I-targeting DNA aptamer with a combined G-quadruplex/duplex architecture and anticoagulant activity [45]. The 58-nucleotide $2^{\prime }$-fluoro-pyrimidine-modified RNA aptamer R9D-14T similarly recognizes (pro)Exosite I, inhibits thrombin generation and thrombin activity, and can be neutralized by a reverse-complement (RC) oligonucleotide [46]. The DNA aptamer M08 likewise demonstrated nanomolar thrombin affinity, anticoagulant activity, and rapid neutralization by a reverse-complement [47]. Apta-1 provides a particularly relevant recent comparison. This 2$2^{\prime }$-fluoro-modified RNA aptamer was reported to preferentially interact with thrombin Exosite II and to inhibit thrombin-dependent PAR1/PAR4 platelet signaling. Importantly, Apta-1 displayed an antithrombotic rather than conventional anticoagulant profile, reducing thrombosis without prolonging bleeding time or producing the clotting-time prolongation typically associated with direct thrombin inhibition [48]. This profile differs from AYA1809002, which primarily targets Exosite I and produces a pronounced anticoagulant effect through inhibition of thrombin-mediated fibrinogen cleavage. Other thrombin aptamer designs have combined multiple thrombin-binding surfaces. HD22 recognizes Exosite II, whereas the bivalent DNA aptamer HD1–22 combines the Exosite I-binding HD1 and Exosite II-binding HD22 modules, resulting in high-affinity thrombin binding and enhanced anticoagulant activity [49]. Within this landscape, AYA1809002 exhibits a distinct combination of properties: Exosite I-directed binding, strong inhibition of thrombin-mediated fibrinogen cleavage while preserving catalytic activity toward small peptide substrates, inhibition of thrombin-dependent platelet activation, preservation of thrombomodulin-dependent protein C activation, and rapid sequence-specific reversal by a reverse-complement (RC) oligonucleotide. These findings position AYA1809002 as a mechanistically distinct thrombin-targeting aptamer, although direct head-to-head studies will be required to establish comparative pharmacological advantages.

### 3.9. Study Limitations and Future Directions

Several limitations of the present study should be acknowledged. This investigation establishes the biochemical, mechanistic, and in vitro functional properties of AYA1809002. Although AYA1809002 demonstrated high-affinity recognition of rat thrombin and retained anticoagulant activity in rat plasma, these findings establish cross-species biochemical and functional compatibility but do not define systemic exposure, circulation half-life, clearance, in vivo pharmacodynamic effects, or antithrombotic efficacy. In particular, in vivo pharmacokinetic and pharmacodynamic studies have not yet been performed. Similarly, although palmitate conjugation preserved thrombin binding, anticoagulant activity, and sequence-specific reversal, its potential pharmacokinetic benefit remains to be established experimentally, as albumin association, systemic exposure, clearance, and pharmacokinetic half-life were not evaluated in the present study. The selectivity panel encompassed multiple coagulation, anticoagulant, and fibrinolytic targets; however, additional coagulation factors not directly evaluated, including FVIIa and FXIIIa, warrant dedicated characterization to further define the selectivity profile of AYA1809002. Accordingly, the present findings should be interpreted within the scope of the in vitro biochemical and functional studies performed here and not as evidence of in vivo efficacy or clinical readiness. Future studies should include dedicated pharmacokinetic and pharmacodynamic characterization, dose–response and duration-of-action studies, assessment of the reversibility of anticoagulant activity in vivo, evaluation of efficacy in relevant thrombosis models, and comprehensive safety and toxicology studies. Collectively, these investigations will be necessary to determine whether the favorable biochemical and in vitro functional properties of AYA1809002 translate into predictable systemic exposure, therapeutically meaningful anticoagulant efficacy, and an adequately controlled safety profile in vivo.

## 4. Materials and Methods

### 4.1. Reagents and Buffers

The DNA aptamer AYA1809002 (5′-GCGGTGTTGCTGGGGTTGGGAGGTTGGAGGAAGCAATCGC-3′), the corresponding reverse-complement (RC) strand (5′-GCGATTGCTTCCTCCAACCTCCCAACCCCAGCAACACCGC-3′), biotinylated and DBCO-modified variants, and control DNA aptamers(GCGCGGTCCCGATTTGGTGTAAAATTCCCTCAGCCCTACA) were synthesized and HPLC-purified by Integrated DNA Technologies (IDT, Coralville, IA, USA), and prepared in nuclease-free water at a final concentration of 100 µM. Human α-thrombin was purchased from Thermo Fisher Scientific (Waltham, MA, USA; Cat. No. RP-43100). The RC antidote oligonucleotide used throughout this study was an unmodified DNA sequence with a standard phosphodiester backbone; no nuclease-resistant chemical modifications were incorporated. Throughout this manuscript, the term “thrombin” refers to human α-thrombin unless otherwise specified. Human γ-Thrombin was purchased from Fisher Scientific (Waltham, MA, USA; Cat. No. 50-883-502). Native rat thrombin was obtained from Abcam (Cambridge, UK; Cat. No. ab185262). Citrated pooled human plasma (blood-derived) was purchased from Innovative Research (Novi, MI, USA; Cat. No. IPLAWBNAC50ML). Innovative Grade U.S. origin Sprague–Dawley rat citrated plasma was obtained from Innovative Research (Cat. No. IGRTSDPLANAC25ML). Unless otherwise specified, phosphate-buffered saline (PBS) used throughout this study consisted of 137 mM NaCl, 2.7 mM KCl, 10 mM Na_2_HPO_4_, 1.8 mM KH_2_PO_4_, pH 7.4. All reagents were analytical grade and used as received.

### 4.2. Biolayer Interferometry (BLI) Binding Analysis

Binding kinetics of AYA1809002 to human α-thrombin and native rat thrombin were determined by biolayer interferometry (BLI) using a GatorPrime instrument (Gator Bio, Palo Alto, CA, USA). Biotinylated AYA1809002 (Bi-AYA1809002) was immobilized onto streptavidin (SA) biosensors (Cat. No. 160002, Gator Bio) using a 25 nM aptamer loading solution. Following baseline equilibration in assay buffer, biosensors were exposed to increasing concentrations of thrombin, followed by dissociation in assay buffer.

For analysis of human α-thrombin (Thermo Fisher Scientific, Cat. No. RP-43100), biosensors were incubated with thrombin at 12.5, 25, and 50 nM, together with a 0 nM reference control. Association and dissociation were monitored for 600 s each. For analysis of native rat thrombin (Abcam, Cambridge, UK; Cat. No. ab185262), biosensors were incubated with thrombin at 3.125, 6.25, 12.5, 25, 50, and 100 nM under identical experimental conditions, with 600 s association and 600 s dissociation phases.

Sensorgrams were reference-subtracted and analyzed using GatorOne software (version 2.15.5.1221, Gator Bio). Binding curves were fitted using a 1:1 Langmuir interaction model to determine the apparent association rate constant (k_*on*_), dissociation rate constant (k_*off*_) and apparent equilibrium dissociation constant (K_*D*_). Human thrombin kinetic parameters were calculated from the 12.5–50 nM concentration range, which provided robust concentration-dependent binding suitable for kinetic analysis. Rat thrombin kinetic parameters were determined using the complete concentration series. All experiments were performed according to the manufacturer’s recommendations.

### 4.3. Enzyme-Linked Competition Binding Assay of Biotinylated Aptamer to Human α- and γ-Thrombin Proteins

High-binding 96-well plates (Nunc MaxiSorp, Thermo Fisher Scientific, Waltham, MA, USA; Cat. No. 44-2404-21) were coated with human α-thrombin (Thermo Fisher Scientific, Cat. No. RP-43100) or human γ-thrombin (Fisher Scientific, Cat. No. 50-883-502) diluted in carbonate–bicarbonate coating buffer (50 mM, pH 9.6; 100 µL/well; coating 5 µg/mL) and incubated overnight at 4 °C. Plates were washed 3× with PBST (PBS, pH 7.4 + 0.05% Tween-20) and blocked with 2% (*w*/*v*) BSA in PBS (200 µL/well, 1 h, room temperature).

Biotinylated AYA1809002 (Bi-AYA1809002) was heat-refolded immediately before use at 95 °C for 3–5 min, slow-cool to room temperature, then equilibrate in binding buffer (PBS, supplemented with 1 mM MgCl_2_, 0.1% BSA and 0.05% Tween-20) and added to thrombin-coated wells at the concentration 20 nM either alone (reference condition) or together with a 100-fold molar excess of competitor (final competitor concentration 2 µM), 100 µL/well. Competitors included unlabeled AYA1809002, reference aptamer NU172, hirudin, dabigatran, PPACK, unfractionated heparin (UFH), and fibrinogen. Mixtures were incubated for 1 h at room temperature with gentle shaking.

Following incubation, wells were washed 4–5 times with PBST to remove unbound material. Bound Bi-AYA1809002 was detected using streptavidin–HRP (Thermo Fisher Scientific, Cat. No. 21130) diluted in binding buffer (100 µL/well) and incubated for 40 min at room temperature. Plates were washed three times with PBST prior to development with 3,3′,5,5′-tetramethylbenzidine (TMB) substrate (Thermo Fisher Scientific, Cat. No. 34021; 100 µL/well). The reaction was stopped with 1 M H_2_SO_4_ (100 µL/well) once signal development was within the linear range. Absorbance was measured at 450 nm with optional reference subtraction at 570–620 nm.

Data were background-corrected (no-aptamer control) and expressed as percent binding relative to Bi-AYA1809002 alone (set to 100%). Each condition was run in technical replicates (≥3 wells/condition), and results are reported as mean ± SD.

### 4.4. Thrombin Catalytic Activity Assay

Thrombin enzymatic activity was measured using a thrombin substrate III, fluorogenic peptide (Sigma-Aldrich, St. Louis, MO, USA; Cat. No. 605211-25MG). Human α-thrombin was incubated with increasing concentrations of AYA1809002 (0–10 µM) for 10 min at 37 °C prior to substrate addition. Upon addition of the fluorogenic substrate, plates were placed into SYNERGY HTX multi-mode reader (BioTek, Agilent Technologies, Santa Clara, CA 95051, USA) set to 37 °C, where they were gently shaken for 30 s to ensure homogeneous mixing. Fluorescence was then recorded kinetically at 3-min intervals over a 30-min period (excitation 360 nm; emission 440 nm). Percent activity was calculated relative to untreated thrombin controls. Dabigatran was used as a positive control inhibitor.

### 4.5. Fibrinogen Kinetic Analysis and IC_50_ Determination

Functional inhibition of thrombin-mediated fibrinogen cleavage was assessed using a thrombin time (TT)-based clotting assay performed on a Siemens CS-5100 automated coagulation analyzer (Siemens Healthineers, Marburg, Germany). For kinetic analysis, varying concentrations of fibrinogen substrate were evaluated under initial-rate clotting conditions to determine the apparent Michaelis–Menten constant (K_*m*_). Reaction parameters were fitted using nonlinear regression to the Michaelis–Menten equation to derive the apparent K_*m*_ value. For IC_50_ determination, citrated human plasma was incubated with increasing concentrations of AYA1809002 prior to addition of thrombin reagent according to the TT assay protocol. Clotting times were recorded automatically by the Siemens CS-5100 optical detection system. Percent inhibition was calculated relative to untreated plasma controls, and IC_50_ values were obtained by nonlinear regression analysis of dose–response curves. The inhibition constant (K_*i*_) was calculated using the Cheng–Prusoff equation: K_*i*_ = (IC_50_)/(1 + ([S])/K_*m*_), where [S] represents the fibrinogen concentration used in the assay [50].

### 4.6. In Vitro Plasma Coagulation Activity Assays

Undiluted citrated plasma was loaded directly onto the CS-5100 automated coagulation analyzer without manual predilution. Prothrombin time (PT), thrombin time (TT), activated partial thromboplastin time (aPTT), Fibrinogen, and individual coagulation factor activities were measured using the corresponding Siemens clinical coagulation assays according to the manufacturer’s validated protocols; any assay-specific sample dilution, when required, was performed automatically by the instrument using the corresponding Siemens diluent.

All assays were conducted using pooled normal human citrated plasma under conditions recommended by the manufacturer, unless otherwise specified.

### 4.7. Biochemical Selectivity Profiling of AYA1809002

The biochemical selectivity of AYA1809002 was evaluated against Factor Xa (FXa), Factor XIa (FXIa), Factor XIIa (FXIIa), plasma kallikrein, activated protein C (APC), plasmin, trypsin, Factor VIII (FVIII), and Factor IX (FIX). AYA1809002 was tested at 0.5, 1, 2, 5, and 10 µM. FXa, plasma kallikrein, plasmin, and trypsin activities were measured using AnaSpec (Fremont, CA, USA) fluorescence-based assay kits (AS-72208, AS-72255, AS-72125, and AS-71124, respectively) according to the manufacturer’s protocols, with final enzyme concentrations/activities of 1.0 µg/mL FXa, 100 ng/mL plasma kallikrein, 100 ng/mL plasmin, and 5 U/well (50 U/mL) trypsin. FXIa (Enzyme Research Laboratories, South Bend, IN, USA; HFXIa 1111a; 6 nM) and FXIIa (HFXIIa 1212a; 0.5 µg/mL) activities were measured using S-2366 (0.5 mM) and S-2302 (0.5 mM), respectively, with AEBSF (0.5 mM) as a positive inhibition control. APC (Thermo Fisher Scientific, RP-43095; 0.5 µg/mL) activity was measured using S-2366 (0.6 mM). Chromogenic activity was monitored kinetically at 405 nm at 37 °C. FVIII and FIX activities were measured using BIOPHE N^TM^ FVIII (A221402-RUO) and Factor IX (A221802-RUO) Chromogenic Assays (HYPHEN BioMed, Neuville-sur-Oise, France), respectively, according to the manufacturer’s instructions. For both assays, citrated human plasma was diluted 1:25, and 50 µL of diluted plasma was used per reaction. Enzyme activities were normalized to the corresponding untreated controls (100%).

For all assays, activity measured in the presence of AYA1809002 was normalized to vehicle-treated controls and expressed as percent activity. Data are reported as mean ± SD from at least three independent experiments.

### 4.8. Thrombomodulin-Dependent Protein C Activation Assay

The effect of AYA1809002 on thrombomodulin-mediated activation of protein C was evaluated using a purified protein system. Human protein C (Thermo Fisher Scientific, Cat. No. HCPC-0080) was activated by human α-thrombin (Thermo Fisher Scientific, Cat. No. RP-43100) in the presence of recombinant human thrombomodulin (PeproTech, Cranbury, NJ, USA; Cat. No. 100-58). Activation reactions were performed in 50 mM Tris-HCl, 125 mM NaCl, pH 7.8, containing 0.1% bovine serum albumin (BSA). Human protein C (2.5 µg/mL) was incubated with human α-thrombin (1 nM) and thrombomodulin (10 nM) for 30 min at 37 °C in the presence or absence of AYA1809002 and comparator inhibitors, in a final reaction volume of 100 µL. AYA1809002 was tested at final concentrations of 0.5, 1, 2, and 5 µM. For antidote reversal experiments, the reverse-complement (RC) oligonucleotide was included at a 1:1 molar ratio relative to AYA1809002. Comparator inhibitors included dabigatran (1 µM), bivalirudin (2 µM), and hirudin (50 nM). Following the 30-min activation period, residual thrombin activity was quenched with hirudin (50 nM), after which Spectrozyme^®^ PCa (Sekisui Diagnostics, Burlington, MA, USA) was added to quantify the activity of generated APC. Substrate hydrolysis was monitored kinetically at 405 nm using Synergy HTX multi-mode microplate reader. Protein C incubated with thrombin and thrombomodulin in the absence of inhibitors was defined as 100% APC generation. Control reactions included protein C alone, protein C plus thrombin, and protein C plus thrombomodulin to verify thrombomodulin-dependent protein C activation. Recombinant human serpin (rhSerpin) was included as a positive control for inhibition of APC chromogenic activity. Results were expressed as percentage APC generation relative to the protein C + thrombin + thrombomodulin control. Each condition was performed in triplicate, and data are presented as mean ± SD.

### 4.9. Platelet Aggregation Assay (Microplate Turbidimetry)

The effect of AYA1809002 on platelet aggregation was assessed using a turbidity-based microplate assay. Whole human blood was collected into EDTA tubes and processed within 1 h of collection. Platelet-rich plasma (PRP) was prepared by centrifugation at 300× *g* for 15 min at room temperature without brake. The upper PRP layer was carefully transferred to a clean tube without disturbing the buffy coat.

For aggregation measurements, 150 µL of PRP was dispensed into each well of a clear, flat-bottom 96-well microplate. Agonist solutions (50 µL) were prepared separately and, where indicated, pre-incubated with AYA1809002 or dabigatran for 30 min at room temperature prior to addition to PRP. AYA1809002 was used at a final concentration of 1 µM. Thrombin-dependent platelet aggregation was induced by addition of human α-thrombin (final concentration 0.25–0.3 U/mL; Prolytix, Essex Junction, VT, USA; HCT-0020). Thrombin-independent aggregation was induced using adenosine diphosphate (ADP; 20 µM), collagen from equine tendon type I (15 µg/mL; Fisher Scientific, Cat. No. NC9533954), the thromboxane A_2_ receptor agonist U46619 (15 µM; Cayman Chemical, Ann Arbor, MI, USA; Cat. No. 16450), PAR1-activating peptide SFLLRN (20 µM; Sigma-Aldrich, St. Louis, MO, USA; Cat. No. S1820), or PAR4-activating peptide AYPGKF (200 µM; Sigma-Aldrich, St. Louis, MO, USA; Cat. No. A3227). In thrombin-containing conditions, the fibrin polymerization inhibitor Gly-Pro-Arg-Pro (GPRP) was included to prevent fibrin clot formation.

Immediately following agonist addition, plates were placed into a temperature-controlled Synergy HTX multimode plate reader set to 37 °C, and absorbance was monitored at 650 nm. Prior to the first reading, plates were shaken for 10 s inside the instrument to ensure uniform mixing. Kinetic measurements were then acquired with a 30-s interval between reads, including 2 s of orbital shaking immediately before each measurement. A total of 19 kinetic reads were collected per well. Each experimental condition was measured in technical triplicate wells per donor. Experiments were repeated using PRP prepared from at least two independent donors. Changes in optical density over time were used as an indicator of platelet aggregation and expressed as percentage aggregation relative to baseline. Data are presented as mean ± SD.

### 4.10. Kinetics of Antidote-Mediated Reversal of AYA1809002 Anticoagulant Activity Assessed by Thrombin Time

Reversal of AYA1809002 anticoagulant activity was evaluated using a thrombin time (TT) kinetic assay on a Siemens CS-5100 coagulation analyzer (Siemens Healthineers, Marburg, Germany). Pooled normal human citrated plasma was equilibrated to assay temperature and incubated with AYA1809002 (final concentration 1 µM) for 1 h to establish steady-state anticoagulant activity. Reversal was initiated by addition of a reverse-complement oligonucleotide (RC) at a 1:1 molar ratio relative to AYA1809002. Samples were gently mixed immediately following RC addition. At 0, 5, 10, 20, 40, 60, and 80 min after antidote addition, aliquots were removed and thrombin time (TT) was measured in triplicate using the manufacturer’s TT reagent protocol on the CS-5100 system. Results were normalized to untreated plasma (no aptamer control) and expressed as percent change in TT relative to control: Percent TT increase = (TT_*sample*_− TT_*control*_)/TT_*control*_ × 100. Data are presented as mean ± SD.

### 4.11. Durability and Antidote-Mediated Reversal of AYA1809002 Anticoagulant Activity After Prolonged Plasma Exposure

The reversibility of AYA1809002 anticoagulant activity following prolonged plasma exposure was evaluated using a reverse-complement (RC) oligonucleotide. Pooled normal human citrated plasma was incubated with AYA1809002 at a final concentration of 1 µM for defined durations (1, 2, 4, 7, and 24 h) at room temperature. Samples were maintained under static conditions during the incubation period. At each time point, the RC was added directly to the plasma at a 1:1 molar ratio relative to AYA1809002 and mixed gently by pipetting. Following RC addition, samples were incubated for an additional 1 h at room temperature to allow oligonucleotide hybridization and neutralization of aptamer activity.

After completion of the post-RC incubation, coagulation assays were performed using a Siemens CS-5100 coagulation analyzer (Siemens Healthineers, Marburg, Germany). Prothrombin time (PT) was measured using Innovin^®^, activated partial thromboplastin time (aPTT) using Actin^®^ FS reagent, thrombin time (TT) using Siemens Thrombin Reagent, and Factor II activity using a clot-based assay with Factor II–deficient plasma. All assays were conducted according to the manufacturer’s instructions. For each incubation time point, plasma samples were tested under three conditions: plasma without aptamer (No Apt), plasma incubated with AYA1809002 alone, and plasma incubated with AYA1809002 followed by RC addition. Coagulation parameters were recorded and expressed as percent change relative to plasma without aptamer, which was defined as 100%.

### 4.12. Functional Stability of AYA1809002 Following Human Serum Exposure

The serum stability and nuclease resistance of AYA1809002 were evaluated using a functional thrombin time (TT) assay following pre-incubation of aptamers in human serum. Human serum was prepared from pooled normal human blood and equilibrated to 37 °C prior to use. AYA1809002 and the reference thrombin aptamer NU172 were incubated separately in human serum at 37 °C for the indicated durations (0, 7, and 24 h).

To assess nuclease sensitivity, parallel serum incubations were performed in the presence of the nuclease inhibitor aurintricarboxylic acid (ATA; Sigma-Aldrich, Cat. No. 1894000), which was added to serum prior to aptamer exposure. Control incubations were conducted in serum without ATA. All serum incubations were carried out under static conditions at 37 °C.

At each time point, aliquots of the serum–aptamer mixtures were transferred into pooled normal human citrated plasma to achieve a final aptamer concentration of 1 µM for functional testing. Thrombin time (TT) was then measured using a Siemens CS-5100 coagulation analyzer (Siemens Healthineers, Marburg, Germany) with Siemens Thrombin Reagent, according to the manufacturer’s instructions. TT values were recorded in seconds and expressed as percent TT increase relative to plasma without aptamer (“None”), which was defined as 100%. Each condition was measured in technical triplicate, and experiments were repeated using independent serum preparations. Data are reported as mean ± SD.

### 4.13. Stability of DNA Aptamers in Simulated Gastrointestinal and Oral Environments

AYA1809002, NU172, and control DNA aptamers were synthesized by Integrated DNA Technologies (IDT), HPLC-purified, and prepared in nuclease-free water at a final concentration of 1 µM unless otherwise indicated.

#### 4.13.1. Gastric-Phase Stability

DNA aptamers were incubated for 1 h at 37 °C in phosphate-buffered saline (PBS) or native porcine gastric fluid (Animal Technologies, Tyler, TX, USA; Cat. No. 221). Following incubation, samples were mixed with denaturing loading buffer and analyzed by denaturing 15% TBE–urea polyacrylamide gel electrophoresis (PAGE) to assess molecular integrity.

#### 4.13.2. Intestinal-Phase Nuclease Challenge

For simulated intestinal-phase conditions, samples were first neutralized to near-physiological pH and then incubated for 1 h at 37 °C with buffer alone, pancreatin (Sigma-Aldrich, Cat. No. P3292-25G), or DNase I (Thermo Fisher Scientific, Cat. No. 18068015) at 2 U/mL, used as a nuclease-enriched stress condition. Reactions were terminated by addition of denaturing PAGE loading buffer and analyzed by denaturing 15% TBE–urea PAGE.

#### 4.13.3. Oral-Phase Stability in Human Saliva

Stability in the oral environment was assessed by incubating AYA1809002 and NU172 in native pooled donor human saliva at 37 °C for up to 60 min without DNase supplementation. Aliquots were collected at the indicated time points and analyzed by denaturing 15% TBE–urea PAGE.

#### 4.13.4. Gel Analysis

Samples were resolved on 15% Mini-PROTEAN^®^ TBE–Urea precast polyacrylamide gels (Bio-Rad Laboratories, Hercules, CA, USA; Cat. No. 456-6053). A 20/100 nt DNA ladder (Integrated DNA Technologies, IDT, Coralville, IA, USA; Cat. No. 51-05-15-02) was included as a molecular size reference. Following electrophoresis, gels were stained with SYBR^TM^ Gold nucleic acid stain (Thermo Fisher Scientific, Cat. No. S11494) according to the manufacturer’s instructions and imaged using a Bio-Rad ChemiDoc^TM^ Touch Imaging System. Aptamer integrity was evaluated based on the presence of single, discrete full-length bands, absence of lower-molecular-weight fragments, and lack of smearing relative to PBS-treated controls.

### 4.14. Accelerated Thermal Stability Study of AYA1809002

Solution-state accelerated stability testing was performed under controlled thermal conditions. AYA1809002 (1 µM in PBS, pH 7.4) was incubated in sealed microcentrifuge tubes at 50 °C or 60 °C using a Hybex Microsample Incubator (SciGene, Sunnyvale, CA, USA). Samples were protected from light throughout the incubation period. To allow simultaneous collection of all time points, sample incubation was initiated in a staggered fashion such that the 4-week, 2-week, and 1-week samples were started at intervals of 2 weeks apart, enabling concurrent termination and analysis of all conditions on a single designated analysis date.

At each designated time point, aliquots were removed simultaneously and allowed to equilibrate to room temperature prior to functional analysis. Residual anticoagulant activity following thermal stress was evaluated using a thrombin time (TT) assay performed on a Siemens CS-5100 coagulation analyzer (Siemens Healthineers, Erlangen, Germany). Results were expressed as percent increase in TT relative to untreated plasma (set to 100%). Untreated plasma served as a negative control, and freshly prepared AYA1809002 (1 µM) served as a positive control. All measurements were performed in triplicate and are reported as mean ± standard deviation (SD). Equivalent shelf-life at 25 °C was estimated using the Q10 model under two assumptions (Q10 = 2 and Q10 = 3) to project temperature-dependent degradation kinetics according to established Arrhenius-based stability modeling principles.

### 4.15. pH Stability Assessment

AYA1809002 (1 µM) was incubated in buffered solutions adjusted to pH 5.0 (50 mM sodium acetate buffer (NaOAc), 150 mM NaCl), pH 7.4 PBS (137 mM NaCl, 2.7 mM KCl, 10 mM Na_2_HPO_4_, 1.8 mM KH_2_PO_4_), or pH 9.0 (50 mM Tris-HCl, 150 mM NaCl) for 1 week at room temperature. Buffer ionic strength was maintained constant across all conditions. Following incubation, samples were returned to neutral PBS (pH 7.4) prior to functional testing to eliminate pH-dependent assay artifacts. Residual anticoagulant activity was evaluated by TT assay on a Siemens CS-5100 coagulation analyzer (Siemens Healthineers, Erlangen, Germany) using pooled normal citrated human plasma. Results were expressed as percent increase in TT relative to untreated plasma (set to 100%). All measurements were performed in triplicate and are reported as mean ± standard deviation (SD). Untreated plasma and freshly prepared AYA1809002 (1 µM) served as negative and positive controls, respectively.

### 4.16. Photostability Testing

Photostability was evaluated by incubating 1 µM AYA1809002 in PBS buffer, (pH 7.4, 137 mM NaCl, 2.7 mM KCl, 10 mM Na_2_HPO_4_, 1.8 mM KH_2_PO_4_) under two conditions for one week: ambient laboratory light exposure or complete protection from light (foil-wrapped tubes). All samples were maintained at room temperature throughout the exposure period. Following incubation, residual anticoagulant activity was assessed in triplicate by TT assay as described above. Untreated plasma and freshly prepared AYA1809002 (1 µM) served as negative and positive controls, respectively.

### 4.17. Oxidative Stress Challenge

Oxidative stability was assessed by incubating AYA1809002 (1 µM) in PBS containing 0.01% hydrogen peroxide (H_2_O_2_) at room temperature for 1 or 2 weeks. Parallel control samples without H_2_O_2_ were maintained under identical conditions. At the designated time points, aliquots were diluted into pooled citrated plasma to minimize residual oxidant prior to TT analysis. Anticoagulant activity was quantified using the Siemens CS-5100 TT assay and expressed as percent TT increase relative to untreated plasma. For all stress conditions (thermal, pH, oxidative, and photostability), anticoagulant function was assessed by thrombin time prolongation. Measurements were performed in triplicate and are reported as mean ± standard deviation (SD). Freshly prepared AYA1809002 (1 µM) served as a positive control, and untreated plasma served as a negative control.

### 4.18. Synthesis of Palmitic Acid–Modified AYA1809002 (Palmitic-AYA1809002)

#### 4.18.1. Materials

16-Azidohexadecanoic acid was purchased from MedChemExpress (Monmouth Junction, NJ, USA; Cat. No. HY-147309). The 5′-DBCO-AYA1809002-3′-biotin DNA aptamer (40 nt; 5′-DBCO-TEG-AYA1809002-3′-Bi) was synthesized and HPLC-purified by Integrated DNA Technologies (IDT, Coralville, IA, USA). HEPES buffer (25 mM, pH 7.5) and molecular biology–grade DMSO were used for conjugation. Desalting columns (GT-600, 1 mL) were obtained from G-Biosciences (St. Louis, MO, USA; Cat. No. 786-170).

#### 4.18.2. Conjugation and Purification of 16-Azidohexadecanoic Acid to DBCO-AYA1809002

Palmitic acid conjugation was performed via copper-free strain-promoted azide–alkyne cycloaddition (SPAAC). 16-Azidohexadecanoic acid was dissolved in DMSO to 84 mM, and the DBCO-modified aptamer was prepared in nuclease-free water at 500 µM. The reaction mixture consisted of 80 µL DMSO, 6 µL azido-palmitic acid solution (500 nmol), and 94 µL of 25 mM HEPES buffer (pH 7.5), added dropwise at 37 °C. Once the solution became homogeneous, 20 µL of 500 µM DBCO-AYA1809002 (10 nmol) was added to achieve an approximately 50-fold molar excess of azide reagent relative to aptamer, for a total reaction volume of 200 µL. The mixture was incubated at 37 °C for 5 h with intermittent vortexing. Following incubation, the reaction mixture was desalted and buffer-exchanged into 25 mM HEPES (pH 7.5) using GT-600 1 mL desalting columns according to the manufacturer’s instructions. The purified palmitic acid conjugated aptamer (Palmitic-AYA1809002-3′-Bi) was collected and stored at 4 °C for short-term use or −20 °C for long-term storage.

#### 4.18.3. Confirmation of Palmitic-AYA1809002 Conjugation and Affinity Determination

DBCO-AYA1809002 and Palmitic-AYA1809002-3′-Bi were analyzed by denaturing 15% TBE–urea polyacrylamide gel electrophoresis (PAGE) to confirm successful palmitic acid conjugation. Samples were resolved on 15% Mini-PROTEAN^®^ TBE–Urea precast gels (Bio-Rad Laboratories, Cat. No. 456-6053). A 20/100 nt DNA ladder (Integrated DNA Technologies, Cat. No. 51-05-15-02) was included as a molecular size reference.

Following electrophoresis, gels were stained with SYBR^TM^ Gold nucleic acid stain (Thermo Fisher Scientific, Cat. No. S11494) and imaged using a ChemiDoc^TM^ Touch Imaging System (Bio-Rad Laboratories). Conjugation was confirmed by the reduced electrophoretic mobility of Palmitic-AYA1809002 relative to DBCO-AYA1809002. A mixture of DBCO- and Palmitic-AYA1809002 was analyzed to demonstrate distinct band separation.

Binding affinity of Palmitic-AYA1809002 was determined using an ELISA-based competition assay. Human α-thrombin (Thermo Fisher Scientific, cat. No. RP-43100) was immobilized on 96-well plates and blocked prior to use. Increasing concentrations of 5′-Palmitic-AYA1809002-3′-biotin were incubated with immobilized thrombin for 1 h at room temperature.

To define specific binding, parallel wells contained a 100-fold molar excess of non-biotinylated AYA1809002. Following washing, bound aptamer was detected with streptavidin–HRP (Thermo Fisher Scientific, cat. No. 21130), diluted in binding buffer 100 µL/well. After 40 min incubation at room temperature, plates were washed 3× with PBST, developed with TMB substrate (Thermo Fisher Scientific, cat. No. 34021). 100 µL/well until signal was in the linear range, and the reaction was stopped with 1 M H_2_SO_4_ (100 µL/well). Absorbance was read at 450 nm. Specific binding was calculated by subtracting signal obtained in the presence of competitor from total binding. Apparent dissociation constant (K_*D*_) values were determined by nonlinear regression analysis using a one-site binding model.

#### 4.18.4. Functional Activity and Reversibility of Palmitic-AYA1809002

Pooled citrated human plasma was incubated with Palmitic-AYA1809002 at a final concentration of 1 µM for 1 h at room temperature. Unmodified AYA1809002 (1 µM) and plasma without aptamer served as positive and negative controls, respectively.

Where specified, a reverse-complement oligonucleotide (RC; reverse-complement sequence) was added simultaneously with the aptamer at defined molar ratios relative to Palmitic-AYA1809002 (RC:aptamer = 0.5:1, 1:1, and 2:1). Prothrombin time (PT), activated partial thromboplastin time (aPTT), thrombin time (TT), and Factor II (FII) activity were measured using a Siemens CS-5100 automated coagulation analyzer (Siemens Healthineers) according to the manufacturer’s protocols. Results are expressed as percent change relative to untreated plasma (set to 100%). For PT, aPTT, and TT assays, data are reported as percent increase compared with untreated plasma. For FII activity, results are expressed as percent activity relative to untreated plasma.

### 4.19. Rat Plasma Functional Assays

Innovative Grade U.S. origin Sprague–Dawley rat citrated plasma (Innovative Research, Cat. No. IGRTSDPLANAC25ML) was used for all coagulation assays. For dose–response experiments, rat plasma was incubated with AYA1809002 at indicated concentrations (0.5–4 µM) prior to analysis. For reversibility studies, plasma was incubated with AYA1809002 (1 µM), followed by addition of a reverse-complement oligonucleotide (RC) at defined molar ratios (1:1, 2:1, 5:1; RC:aptamer). Samples were equilibrated prior to measurement. Prothrombin time (PT), activated partial thromboplastin time (aPTT), thrombin time (TT), and Factor II (FII) activity were loaded directly onto the CS-5100 analyzer without manual predilution; any assay-specific sample dilution, when required, was performed automatically by the instrument according to the corresponding Siemens assay protocol.

Results are expressed as percent change relative to untreated plasma (set to 100%). For PT, aPTT, TT, and FII assays, data are reported as percent compared with untreated plasma.

### 4.20. Statistical Methods

Statistical analyses were performed using IBM SPSS Statistics (version 31; IBM Inc., Chicago, IL, USA). Data are presented as mean ± standard deviation (SD) from independent experiments. All analyses were performed using the original, non-normalized measurements.

For concentration-dependent effects of the aptamer, a repeated-measures analysis was used to evaluate overall differences across concentrations. Overall differences among treatment conditions were assessed using the Friedman test. When appropriate, prespecified pairwise comparisons were performed using two-sided paired Student’s *t*-tests to compare each concentration or treatment condition with the 0-concentration (None) control or the corresponding untreated condition. *p*-values from multiple pairwise comparisons were adjusted using the Holm method. Statistical significance was defined as a two-sided adjusted *p* < 0.05.

## 5. Conclusions

In conclusion, AYA1809002 is a high-affinity, Exosite I-targeting DNA aptamer that selectively modulates thrombin function while enabling rapid sequence-specific reversal by a reverse-complement (RC) oligonucleotide. The present study establishes its biochemical mechanism, functional anticoagulant activity, selectivity, reversibility, functional stability, and cross-species activity in rat thrombin and rat plasma. These findings support further investigation of AYA1809002 as a selective and controllable thrombin-targeting aptamer. Dedicated in vivo pharmacokinetic, pharmacodynamic, efficacy, and safety studies will be required to determine whether these in vitro properties translate into effective and predictable anticoagulant activity in vivo.

## Figures and Tables

**Table 1:** Biochemical selectivity profile of AYA1809002. Thrombin inhibitory activity was determined using a thrombin time (TT)-based fibrinogen clotting assay. Selectivity against non-thrombin targets was evaluated using purified enzyme or chromogenic activity assays as appropriate. Thrombin IC_50_ was determined from concentration–response analysis, and K_*i*_ was calculated using the Cheng–Prusoff relationship. No measurable inhibition was observed for any non-thrombin target at concentrations up to 10 µM.

Target	Assay Type	IC_50_ (nM)	K_*i*_ (nM)	Maximum Inhibition Observed
Thrombin	Thrombin time (TT) fibrinogen clotting assay	25.7	10.6	>95%
Factor Xa (FXa)	Purified enzyme assay	>10,000	n/a	<10%
Factor XIa (FXIa)	Purified enzyme assay	>10,000	n/a	<10%
Factor XIIa (FXIIa)	Purified enzyme assay	>10,000	n/a	<10%
Plasma kallikrein	Purified enzyme assay	>10,000	n/a	<10%
Activated protein C (APC)	Purified enzyme assay	>10,000	n/a	<10%
Plasmin	Purified enzyme assay	>10,000	n/a	<10%
Trypsin	Purified enzyme assay	>10,000	n/a	<10%
Factor VIII (FVIII:C)	Chromogenic activity assay	>10,000	n/a	No inhibition detected
Factor IX (FIX)	Chromogenic activity assay	>10,000	n/a	No inhibition detected

**Table 2:** Functional stability of AYA1809002 and NU172 following incubation in human serum. Aptamers were incubated in human serum at 37 °C for 7 or 24 h prior to functional testing. Aliquots were transferred to pooled citrated human plasma (final concentration of the aptamers 1 µM) and coagulation parameters were measured: prothrombin time (PT), activated partial thromboplastin time (aPTT), Factor II (FII) activity, and thrombin time (TT). Retained anticoagulant activity is expressed as a percentage relative to each aptamer’s activity at time 0 (set to 100%). Each measurement represents the average of triplicate determinations.

	PT		aPTT		FII		TT	
	AYA1809002	NU172	AYA1809002	NU172	AYA1809002	NU172	AYA1809002	NU172
Retained activity after 7 h incubation with serum proteases	68%	9%	86%	24%	89%	43%	96%	13%
Retained activity after 24 h incubation with serum proteases	36%	0%	45%	0%	60%	10%	53%	6%

**Table 3:** Preliminary Q10-Based Extrapolation of AYA1809002 Accelerated Thermal Stability Data. Equivalent shelf-life at 25 °C was estimated using the Arrhenius Q10 method in accordance with ICH Q1E guidance. Calculations were performed using Q10 values of 2 (conservative estimate) and 3 (commonly applied for oligonucleotide stability modeling). Q10-based values represent theoretical extrapolations from accelerated thermal stress conditions and are provided for exploratory purposes only. They have not been validated by real-time stability studies and should not be interpreted as established shelf life or expiration dating for AYA1809002.

Test Condition	Duration	Theoretical Equivalent Duration at 25°C (Q10 = 2)	Equivalent Shelf-Life at 25 °C (Q10 = 3)
50 °C	1 week	∼1 month	∼3.6 months
50 °C	2 weeks	∼2 months	∼7.2 months
50 °C	4 weeks	∼4 months	∼1.2 years
60 °C	1 week	∼3 months	∼11 months
60 °C	2 weeks	∼6 months	∼1.8 years
60 °C	4 weeks	∼1 year	∼3.6 years

## Data Availability

All data generated or analyzed during this study are included in this published article.

## Supplementary Materials

The following supporting information can be downloaded at: https://www.mdpi.com/article/10.3390/ph19091431/s1, Figure S1: Biochemical selectivity profile of AYA1809002 against coagulation, anticoagulant, fibrinolytic, and related serine proteases. Figure S2. AYA1809002 preserves platelet aggregation induced by additional non-thrombin agonists. Figure S3. Structural stability of AYA1809002 and NU172 in simulated gastrointestinal and oral environments. Figure S4. AYA1809002 maintains anticoagulant activity under pH, light, and oxidative stress conditions. Figure S5. AYA1809002 retains anticoagulant activity under accelerated thermal stress conditions. Table S1: Raw coagulation parameters measured during prolonged plasma incubation in the reversal study. Table S2. Statistical Table for Figure 3B. Table S3. Statistical Table for Figure 4. Table S4. Statistical Table for Figure 9. Table S5. Statistical table for Figure 11.

## Abbreviations

The following abbreviations are used in this manuscript:

PT	Prothrombin time
aPTT	Activated partial thromboplastin time
TT	Thrombin time
FII	Factor II
FXa	Factor Xa
FXIa	Factor XIa
FXIIa	Factor XIIa
APC	Activated protein C
FVIII	Factor VIII
FIX	Factor IX
RC	Reverse-complement
BLI	Biolayer interferometry
TM	Thrombomodulin
PrC	Protein C
PAR	Protease-activated receptor
ADP	Adenosine diphosphate
IC_50_	Half-maximal inhibitory concentration
K_*i*_	Inhibition constant
K_*D*_	Equilibrium dissociation constant
k_*on*_	Association rate constant
k_*off*_	Dissociation rate constant
ATA	Aurintricarboxylic acid
PAGE	Polyacrylamide gel electrophoresis
TBE	Tris-borate-EDTA
SD	Standard deviation
GPRP	Gly-Pro-Arg-Pro
UFH	Unfractionated heparin
PPACK	D-Phe-Pro-Arg chloromethylketone
TMB	3,3′,5,5′-Tetramethylbenzidine
BSA	Bovine serum albumin
DBCO	Dibenzocyclooctyne
SPAAC	Strain-promoted azide–alkyne cycloaddition
TEG	Triethylene glycol
HEPES	4-(2-Hydroxyethyl)-1-piperazineethanesulfonic acid
