# Supplementary material for: Selective Thrombin Modulation by AYA1809002: Stability and On-Demand Reversal of an Exosite I-Targeting Aptamer
Source: Pharmaceuticals (Basel). 2026 Sep 10;19(9):1431. doi: 10.3390/ph19091431 (PMC13610730; doi:10.3390/ph19091431)
Supplement: Supplementary file 1 [file pharmaceuticals-19-01431-s001.zip › supplementary_figure_062626.pdf]

From Discovery to Development: Enhancing Stability and On-Demand Reversal of a Clinical-Candidate Thrombin Aptamer

Supplementary

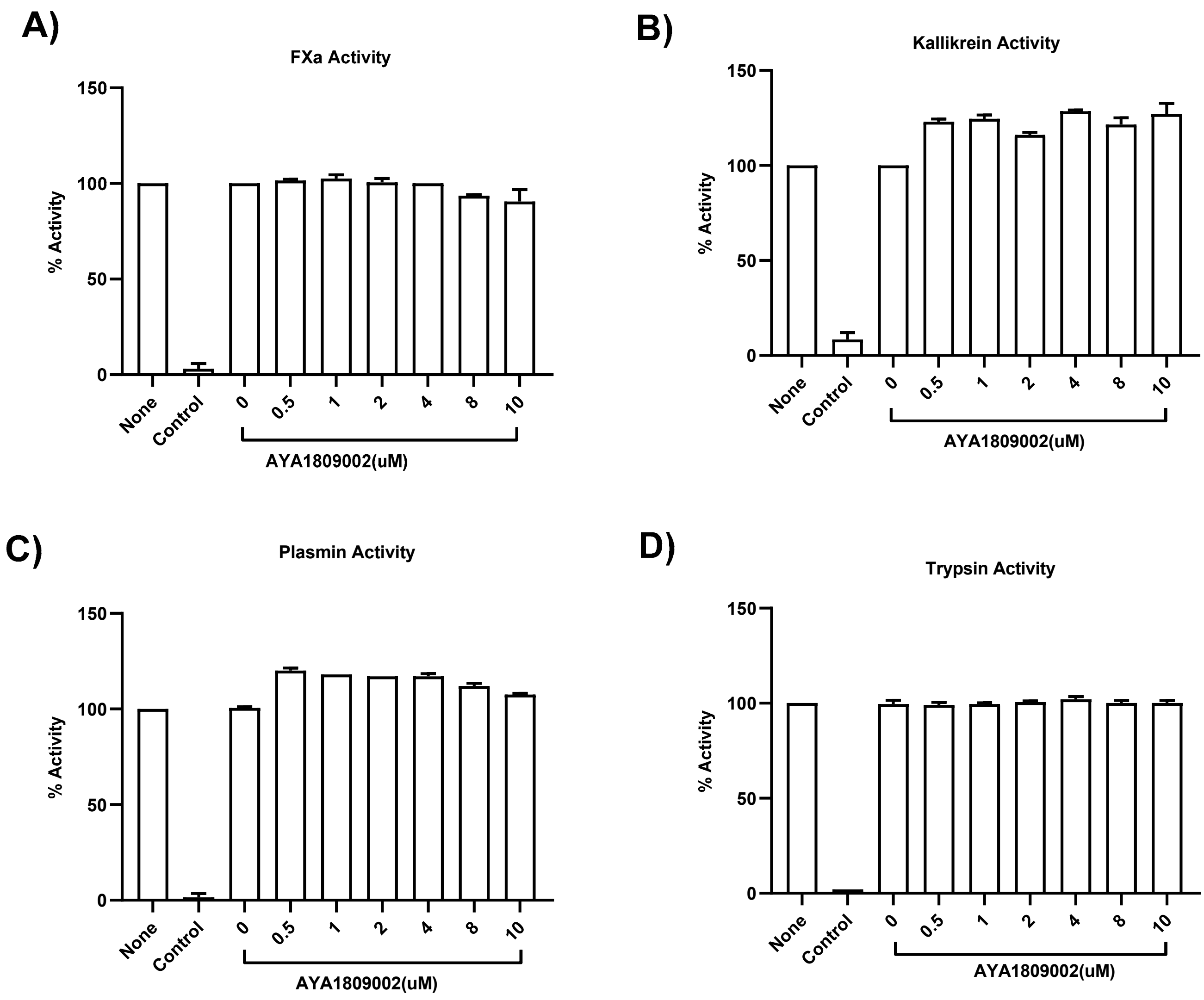

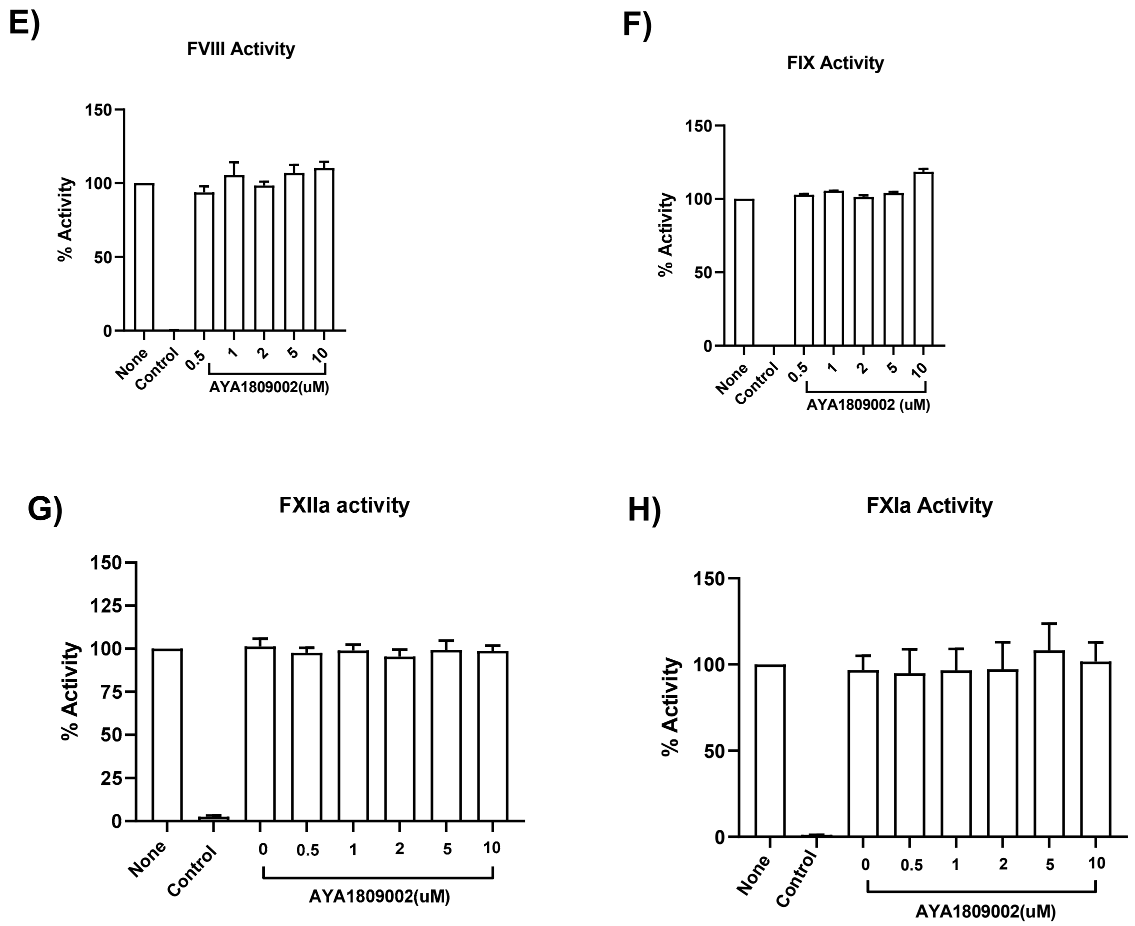
E)
FVIII Activity
% Activity
150
100
50
0
None
Control
0.5
1
2
5
10
AYA1809002(uM)
F)
FIX Activity
% Activity
150
100
50
0
None
Control
0.5
1
2
5
10
AYA1809002 (uM)
G)
FXIIa activity
% Activity
150
125
100
75
50
25
0
None
Control
0
0.5
1
2
5
10
AYA1809002(uM)
H)
FXIa Activity
% Activity
150
100
50
0
None
Control
0
0.5
1
2
5
10
AYA1809002(uM)

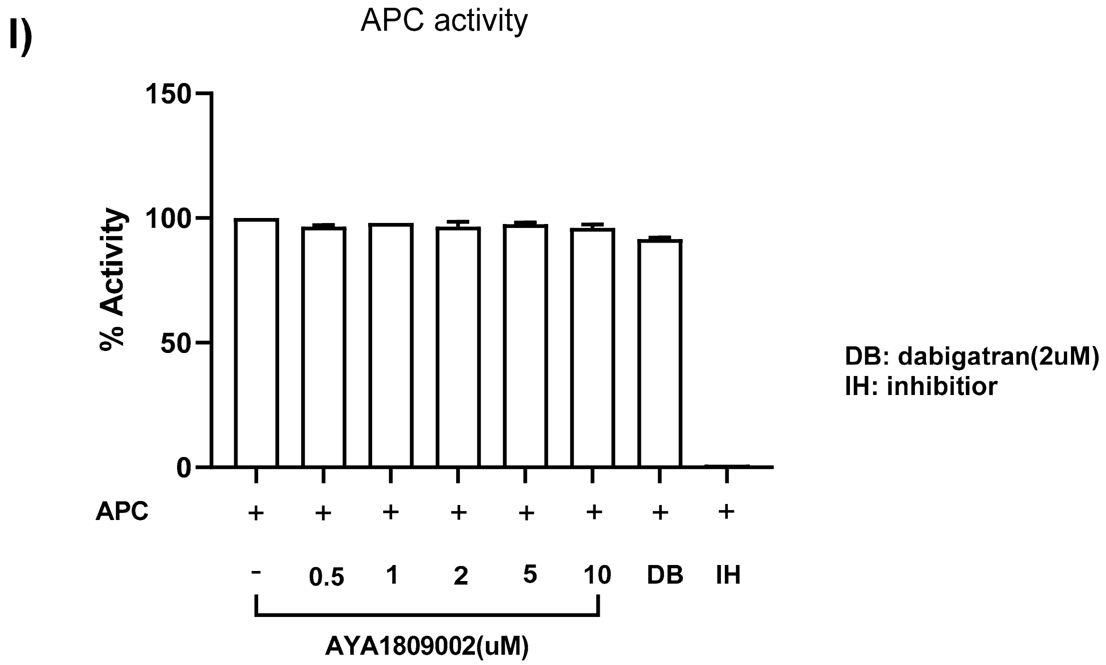

Figure S1. Biochemical selectivity profile of AYA1809002 against coagulation, anticoagulant, fibrinolytic, and related serine proteases. Enzymatic activity assays were performed using purified human proteins in the presence or absence of AYA1809002 (10 µM). The activity of (A) Factor Xa (FXa), (B) plasma kallikrein, (C) plasmin, (D) trypsin, (E) Factor VIII (FVIII), (F) Factor IX (FIX), (G) Factor XIIa (FXIIa), (H) Factor XIa (FXIa), and (I) activated protein C (APC) was measured using the corresponding chromogenic or fluorogenic substrates according to the manufacturer's protocols. Enzyme activity is expressed relative to untreated control reactions (100%). No measurable inhibition was observed for any enzyme tested, demonstrating the high biochemical selectivity of AYA1809002 for thrombin. Data represent mean ± SD of triplicate measurements (N = 3). Statistical analysis showed no significant differences compared with untreated controls (NS).

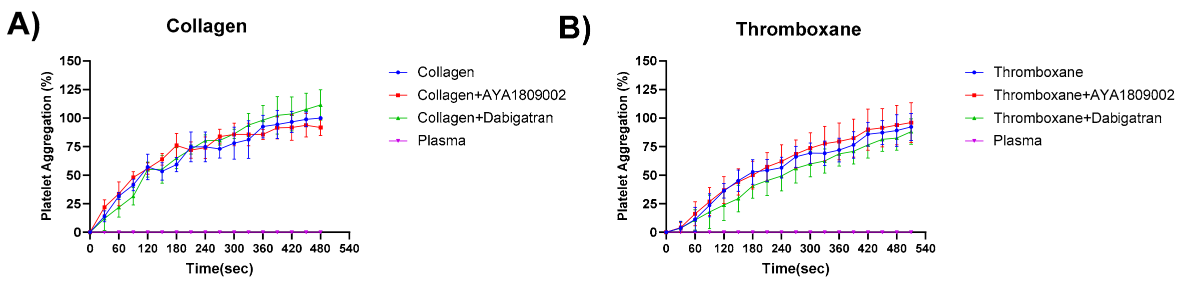

Figure S2. AYA1809002 preserves platelet aggregation induced by additional non-thrombin agonists. Platelet aggregation was measured in human platelet-rich plasma (PRP) by kinetic microplate turbidimetry (OD650) at 37 °C as described in Materials and Methods. Agonists were pre-incubated with AYA1809002 (1 µM) for 30 min at room temperature prior to addition to PRP. (A) Platelet aggregation induced by collagen (15 µg/mL). (B) Platelet aggregation induced by the thromboxane A_2 receptor agonist U46619 (15 µM). AYA1809002 did not inhibit platelet aggregation induced by either agonist, confirming that its antiplatelet activity is selective for thrombin-mediated signaling while preserving platelet responses to non-thrombin agonists. Data are presented as mean ± SD from technical duplicate wells using PRP prepared from at least two independent donors.

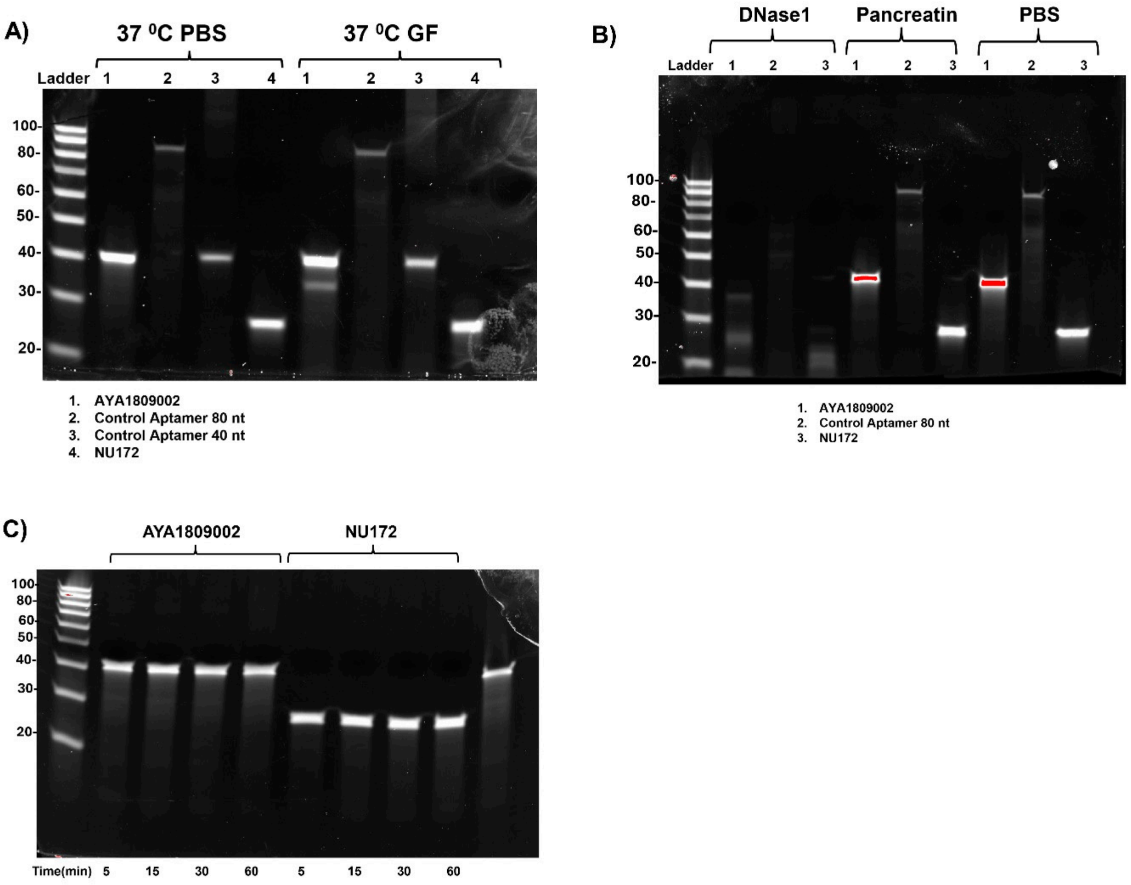

Figure S3. Structural stability of AYA1809002 and NU172 in simulated gastrointestinal and oral environments. (A) AYA1809002, NU172, and control aptamers (1µM) were incubated for 1 hour at 37 ◦C in phosphate-buffered saline (PBS) or native gastric fluid (GF) and analyzed by denaturing 15% TBE–urea polyacrylamide gel electrophoresis (PAGE). All aptamers remained intact following gastric incubation, with no detectable fragmentation or loss of full-length material. (B) Following neutralization to near-physiological pH, aptamers were incubated for 1 hour at 37 ◦C under intestinal-phase conditions including buffer alone, pancreatin, or DNase I (2 U/mL; nuclease-enriched stress condition) and analyzed by denaturing 15% TBE–urea PAGE. DNase I induced rapid degradation of all aptamers, whereas pancreatin produced no detectable effect on aptamer integrity; buffer controls remained intact. (C) Stability in the oral environment was assessed by incubating AYA1809002 and NU172 in native human saliva at 37 ◦C for up to 60 minutes. No detectable degradation, smearing, or reduction in band intensity was observed for either aptamer over the incubation period.

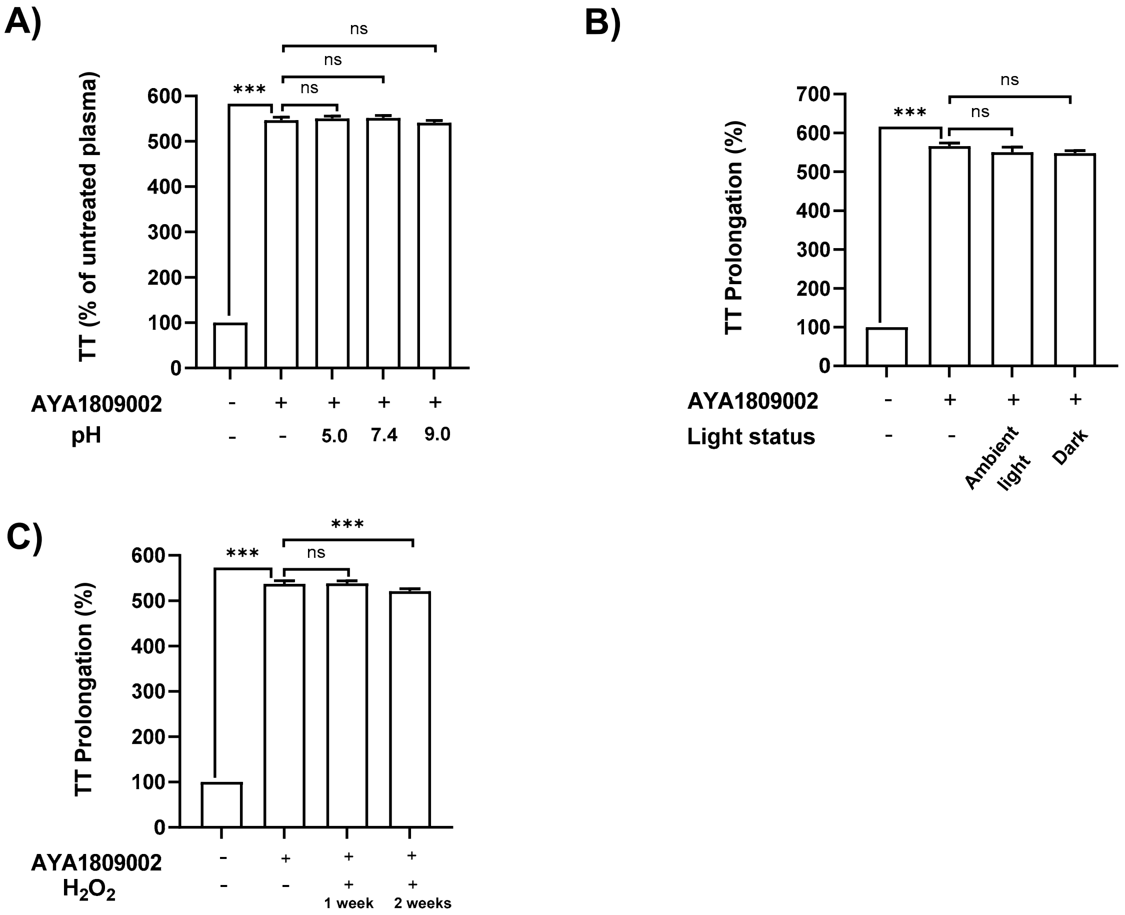

Figure S4. AYA1809002 maintains anticoagulant activity under pH, light, and oxidative stress conditions. (A) AYA1809002 (1 µM) was incubated for 1 week at the indicated pH values (pH 5.0, 7.4, and 9.0) prior to functional assessment. (B) AYA1809002 was stored under ambient laboratory light or dark conditions for 1 week. (C) AYA1809002 was exposed to hydrogen peroxide (0.01% H_2O_2) for the indicated durations (1 or 2 weeks). For all conditions, samples were transferred to pooled citrated human plasma following treatment and thrombin time (TT) was measured. Results are expressed as TT prolongation (%) relative to untreated plasma control. Freshly prepared AYA1809002 was included as a positive control. Across all conditions tested, AYA1809002 retained thrombin-inhibitory activity, indicating robust physicochemical stability. Data represent mean ± SD of triplicate measurements. N=5. *** indicates that p-value<0.001. NS indicates no significant difference.

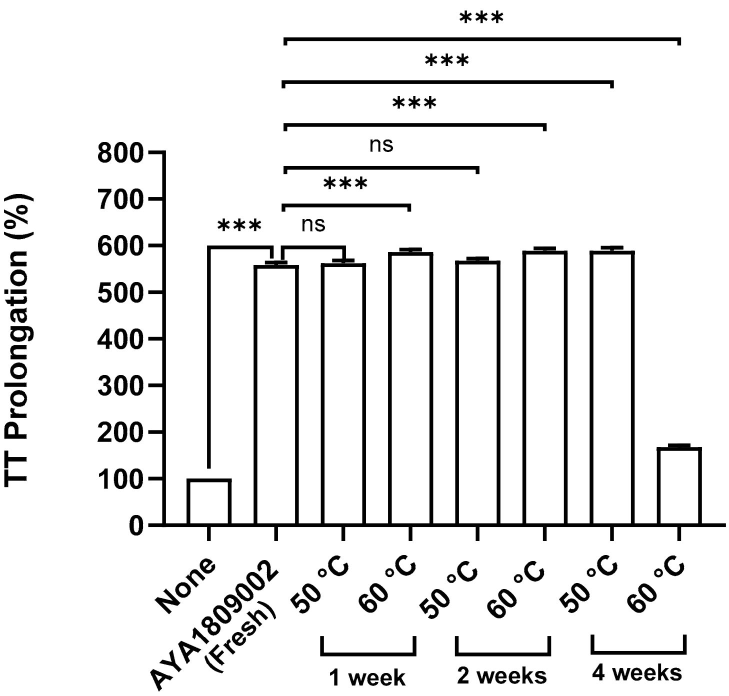

Figure S5 . AYA1809002 retains anticoagulant activity under accelerated thermal stress conditions. AYA1809002 (1 μM) was incubated in buffer at 50 °C and 60 °C for 1, 2, and 4 weeks. Following thermal incubation, samples were returned to room temperature and anticoagulant activity was assessed by thrombin time (TT). Results are expressed as TT prolongation (%) relative to untreated plasma. Untreated plasma and freshly prepared AYA1809002 were included as negative and positive controls, respectively. AYA1809002 retained full anticoagulant activity at both temperatures for up to 2 weeks, with partial loss of activity observed only after 4 weeks at 60 °C. Data represent mean ± SD of triplicate measurements. N=5. *** indicates that p-value<0.001. NS indicates no significant difference.
